# The distribution of initial estimates moderates the effect of social influence on the wisdom of the crowd

**DOI:** 10.1038/s41598-022-20551-7

**Published:** 2022-10-03

**Authors:** Abdullah Almaatouq, M. Amin Rahimian, Jason W. Burton, Abdulla Alhajri

**Affiliations:** 1 Sloan School of Management, Massachusetts Institute of Technology, Cambridge, USA; 2 Department of Industrial Engineering, University of Pittsburgh, Pittsburgh, USA; 3 Department of Digitalization, Copenhagen Business School, Copenhagen, Denmark; 4 Department of Physics, University of Oxford, Oxford, UK

**Keywords:** Psychology, Mathematics and computing

## Abstract

Whether, and under what conditions, groups exhibit “crowd wisdom” has been a major focus of research across the social and computational sciences. Much of this work has focused on the role of social influence in promoting the wisdom of the crowd versus leading the crowd astray and has resulted in conflicting conclusions about how social network structure determines the impact of social influence. Here, we demonstrate that it is not enough to consider the network structure in isolation. Using theoretical analysis, numerical simulation, and reanalysis of four experimental datasets (totaling 2885 human subjects), we find that the wisdom of crowds critically depends on the interaction between (i) the centralization of the social influence network and (ii) the distribution of the initial individual estimates. By adopting a framework that integrates both the structure of the social influence and the distribution of the initial estimates, we bring previously conflicting results under one theoretical framework and clarify the effects of social influence on the wisdom of crowds.

## Introduction

In its classical definition, the concept of “the wisdom of crowds” refers to the idea that the aggregate estimate of a group of individuals can be superior to that of individual, credentialed experts^1,2^. Recent applications of this concept include technological, political, and economic forecasting^3^; performance evaluations^4^; preference elicitation^5^; and public policy design^6^. Conventional statistical accounts of the wisdom of crowds rely on the following two assumptions: (i) the individual errors are uncorrelated or negatively correlated^7^, and (ii) the individuals are unbiased; that is, they are correct in mean expectations^2^.

However, social influence processes, in which people exchange information about their estimates, can cause individuals to revise their judgment in estimation tasks^8–12^. Therefore, a simple averaging of the revised (post-influence) estimates is not the same as simply averaging the initial (pre-influence) estimates. Prior research yields conflicting findings on the effects of social influence on the wisdom of crowds. For instance, despite the evidence that social influence can significantly benefit group and individual estimates^10,12–17^, it has also been found to induce systematic bias, herding, and groupthink^8,9^.

In response to these inconsistencies, notable reconciliation efforts have focused on investigating how social network theories interact with the process of collective belief formation. The results of these efforts, including seminal theoretical works^11,18^ and laboratory experiments^10^, have established that the wisdom of crowds is preserved only if the influence of the most influential individual vanishes (i.e., becomes negligible) as the group size grows^11^. This condition is satisfied in *decentralized influence structures*, wherein everyone has an equal voice, as opposed to *centralized structures* in which one or more individuals have disproportionate influence. Intuitively, the wisdom of crowds benefits from larger group sizes because even if individuals are on average biased, their collective estimate has lower variance; however, centralized influence diminishes this benefit by reducing the collective estimate to the “wisdom of the few.”

While these results appear to broadly suggest the superiority of decentralized influence, their conclusions rest on the premise that the distribution of the initial estimates is centered around the truth. In such situations, there are no opportunities for the crowd to improve with social influence^11^. However, empirical distributions of numerical estimates tend to be right-skewed with excess kurtosis: most estimates are low, with a minority falling on a fat right tail^9,19,20^. The skewness of the distribution could emerge due to systematic bias (a tendency to over- or underestimate the actual value^19,21,22^) or dispersion (the spread of the estimates) in the population. Therefore, it is when the crowd is not initially centered around the truth, as observed in many empirical settings, that centralized influence could present an opportunity to promote crowd wisdom.

In this study, we ask *when centralized influence structures improve or hinder the wisdom of crowds in estimation tasks*. Our results demonstrate that the effect of social influence varies systematically with the distribution of the initial estimates and, therefore, is more heterogeneous than previously suggested. Specifically, we analyze — theoretically, numerically, and empirically — the effect of the distribution of initial estimates on the suitability of a crowd to benefit from influence centralization.

## Theoretical model

**Figure 1:**
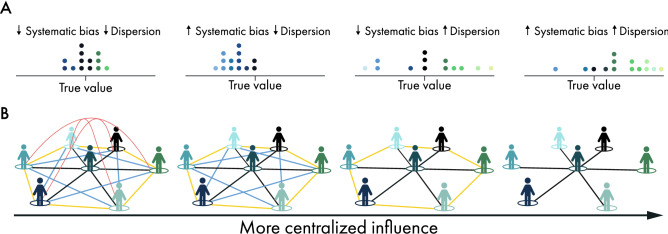
This schematic illustrates our framework for analyzing the role of estimation context in determining how social influence shapes the wisdom of crowds. Panel (**A**) illustrates four distributions of the initial estimates. Panel (**B**) provides examples of different influence network structures arranged in the order of increasing centralization—from a fully decentralized structure, where everyone has an equal voice, to a highly centralized structure, where there is one highly influential individual.

To illustrate this, we consider a group of *n* agents tasked to estimate or forecast, with maximal accuracy, some unknown positive quantity such as the unemployment rate in the next quarter, life expectancy of an ill patient, number of calories in a meal, prevalence of global influenza infections in two weeks, or number of jellybeans in a jar. To model the population of the agents performing a particular estimation task, we endow each agent with an initial estimate based on a biased and noisy signal about the truth. Let the group of *n* agents be indexed by $i = 1, \ldots , n$, and denote their initial estimates by ${\mathbf {a}}_{i,0}$. The initial estimates are independent and identically distributed, and their common distribution, ${\mathcal {F}}_{\mu ,\sigma }^{\theta }$, is parametrized by the unknown truth, $\theta$, the systematic bias, $\mu$, and the dispersion, $\sigma$. The location parameter ($\mu$) indicates the center of the distribution that biases the estimates with respect to the truth, and the shape parameter ($\sigma$) determines the variation and tail shape. The skewness of the distribution can emerge due to several possibly interdependent factors: disproportionate exposure to a skewed sample of the task instance^23,24^, the tendency to over-attend to information that supports one’s hypotheses^25^, or the level of demonstrability of the task at hand^26^. In general, the initial estimates can be viewed as intrinsic properties of the *estimation context*: a population of agents performing a particular estimation task instance. Different populations of agents, such as experts vs. novices, might have different biases and dispersions for the same task instance. Conversely, the same population can vary in terms of their bias and dispersion across different task instances. For brevity and to abstract the agents and the estimation task, we refer to the distribution of the initial estimates as the *estimation context*. Figure 1A shows four estimation contexts with varying levels of bias and dispersion.

Agents frequently have access to the opinions or estimates of other agents. We define the collective estimate of the *n* agents as the average of their revised (post-influence) estimates and denote it by ${{\mathbf {a}}}^{n}$. In many common models of social influence^11,18,27^, as well as in other aggregation mechanisms^7,28,29^, the collective estimate of the group of agents can be expressed as a convex combination (weighted average) of the initial estimates: ${{\mathbf {a}}}^{n}({\bar{w}}) = \sum _{i=1}^{n}w_i{\mathbf {a}}_{i,0}$, where $w_1,\ldots ,w_n$ are positive real weights summing to one. These weights represent the influence of individual agents on shaping the collective estimate. Without loss of generality, we assume that the agents are ordered in decreasing order of their influence so that $w_1 \ge w_2 \ge \cdots \ge w_n$. This definition of collective estimation contains the simple average of the initial estimates—the typical “wisdom of crowds”—as a special case.

We introduce an influence-*centralization* parameter, $\omega$, to interpolate between a collective estimate produced by a fully decentralized influence setup where every agent has an equal voice (i.e., $\omega = 0$ and $w_1 = w_2 = \cdots = w_n = 1/n$) and a dictatorial setup with a single influential agent (i.e., $w_1 =\omega = 1$ and $w_2 = \cdots = w_n = 0$). To investigate the role of network centralization, $0 \le \omega \le 1$, we consider a class of influence structures indexed by $\omega$ such that (see SI Sect. 1.1 for more details),

$$\begin{aligned} {{\mathbf {a}}}^{n}(\omega ) = \omega {\mathbf {a}}_{1,0} + (1-\omega )\frac{1}{n}\sum _{i=1}^{n}{\mathbf {a}}_{i,0}. \end{aligned}$$

Our definition of $\omega$ coincides with Freeman’s centralization^30^ for a class of network typologies that encompass cases of practical and empirical interest, such as fully connected networks, star networks, empty graphs (isolated individuals), and circular lattices, among others. Figure 1B shows four influence network structures in this class (see SI Sect. S1.1 for calculation of $\omega$ in different networks). It is important to note that these networks are *influence networks* (in which a tie between two people is represented as a weighted value between zero and one) and not *communication networks* (i.e., binary networks that define who communicates with whom).

We measure the collective performance of the agents in terms of the proximity of the collective estimate (${{\mathbf {a}}}^{n}$) to the truth ($\theta$). Given the estimation context (the distribution of the initial estimates), our outcome of interest is whether the collective estimate produced by a centralized influence structure outperforms a decentralized baseline. We compute the probability of this outcome for a given estimation context and denote it by $\Omega _n$. Notably, $\Omega _n$ captures a critical *feature* of the estimation context, namely, *its suitability to benefit from centralization*. When $\Omega _n < 1/2$, the initial estimates are better suited for decentralized influence structures; conversely, when $\Omega _n > 1/2$, they are better suited for centralized influence structures.

## Results

### Analytical results

Our theoretical analysis of $\Omega _n$ verifies that for heavy-tailed or right-skewed distributions, the performance of the collective estimate in a centralized structure where a single agent has a non-vanishing influence (their contribution to the collective estimate does not go to zero as $n\rightarrow \infty$) is superior to that of the decentralized baseline. In particular, for heavy-tailed distributions (e.g., Pareto, log-normal, and log-Laplace), we identify phase-transition behaviors, whereby the lower bound’s limiting value transitions from 0 to 1 or 1/2 as the shape parameter, $\sigma$, crosses a critical value (see SI Sect. S2.1). Intuitively, this is because in decentralized networks the sample mean of a heavy-tailed distribution is dominated by its excess tail risk (the egregious errors of a few individuals). On the other hand, with weighted averages as in centralized structures, we can guarantee that some random individuals exert enough influence to prevent the group aggregate from being swayed too far by the flagrant errors of the few. Recall that in our model individuals are equally likely to occupy the central position and, in particular, the people whose opinions are extremely different from the majority are very few and thus correspondingly unlikely to occupy the center. Notably, in this model, centralized structures violate the vanishing influence condition for the wisdom of crowds; see^11^ and SI Sect. S2.15. This underscores the importance of the distributional assumptions, which are context dependent, when studying the effect of social influence on the wisdom of crowds.

**Figure 2:**
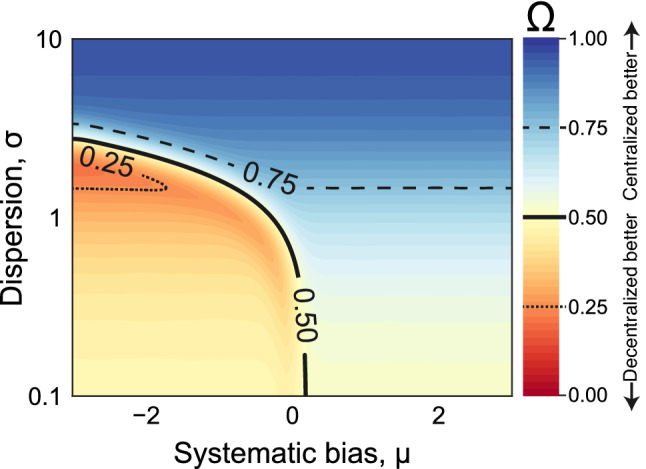
The link between the distribution of the initial estimates and the probability that collective estimation on a centralized structure outperforms a decentralized structure ($\Omega _n$) Our outcome of interest, $\Omega _n$, is the likelihood that a weighted average falls closer to the truth than an unweighted average. Hence, when $\Omega _n < 1/2$, the estimation context is better suited for decentralized (unweighted) influence structures; conversely, when $\Omega _n > 1/2$, it is better suited for centralized (weighted) influence structures. In this figure, the initial estimates are sampled from a log-normal distribution while varying location and shape parameters ($\mu$ and $\sigma$). The number of agents and the influence-centralization level are fixed at $n=50$ and $\omega = 1/3$, respectively. See SI Figs. S1 and S3 for other distributions and parameter choices.

In Fig. 2, we illustrate the behavior of $\Omega _{n}$ for a log-normal distribution of initial estimates, as reported in several empirical studies^9,10,20^. In this case, $\Omega _{n}$ predicts that centralized influence structures improve collective estimates over decentralized ones if the distribution of the initial estimates is characterized by overestimation bias or large dispersion (see SI Sect. S2.2.1 and Fig. S2 for the effect of the systematic bias). However, this relationship is reversed when the initial-estimate distribution is characterized by low dispersion and underestimation bias (see SI Sect.  S2.2 and Fig. S1 for simulation details and other distributional classes).

### Reanalysis of four experimental datasets

**Figure 3:**
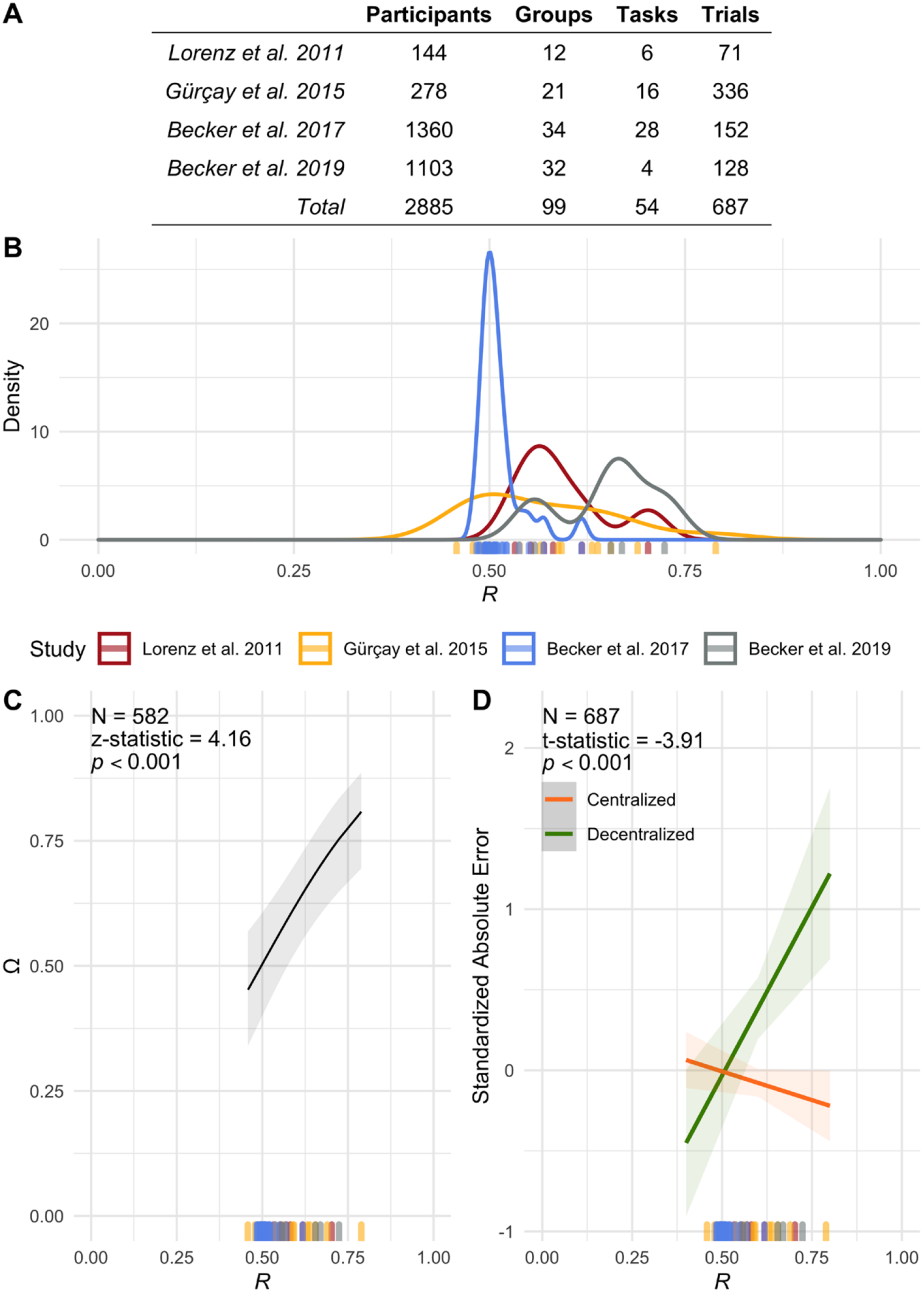
Reanalysis of previously published experiments indicates that our proposed feature, *R*, has significant predictive power for determining when the group performance improves as a result of social interactions. Panel (**A**) shows the number of participants, groups, tasks, and trials in the reanalyzed experiments. Panel (**B**) displays the distribution of *R* across these studies. Panel (**C**) shows that the probability of groups improving their performance after social interaction, $\Omega$, is substantially explained by *R*. Panel (**D**) shows the marginal effect of the interaction term between influence centralization and *R*: as *R* increases, the group performance improves in the centralized influence conditions, and degrades in the decentralized influence conditions. The bands are the $95\%$ confidence intervals.

To empirically test the predictions of the aforementioned model, we use the data pertaining to positive numerical estimation tasks (i.e., tasks with negative estimates omitted) from four published human-subject laboratory experiments^9,10,14,16^. In these experiments, a total of 2885 human participants, organized into 99 independent groups, completed a total of 54 estimation tasks, generating a total of 15,562 individual estimations and 687 collective estimations (see Fig. 3A).

All four experiments followed a similar procedure that involved three steps: (1) the participants simultaneously and independently completed numeric estimation tasks on a range of topics such as visual estimation, trivia questions, political facts, and economic forecasts; (2) within groups of various sizes, the participants in the social interaction condition communicated information about their estimates with each other; and (3) the participants had one or more opportunities to revise their estimates. One trial consisted of a single group of participants answering a single task.

Each task induces a different distribution on the initial estimates that are observed empirically. For each task, we use the relative log likelihood of a fitted log-normal distribution versus a normal distribution (hereinafter denoted by *R*) as a measure of the heavy-tailedness of the initial-estimate distribution. In other words, this measure captures whether the initial estimates for a given task are better described by a thin-tailed distribution (i.e., normal distribution) or a heavy-tailed one (i.e., log-normal distribution). $R=0$ indicates with 100% certainty that the initial estimates are better fit by a normal distribution than a log-normal one, $R=1$ indicates with 100% certainty that the initial estimates are better fit by a log-normal distribution than a normal one, and $R=0.5$ indicates that the initial estimates could equally be described as normally or log-normally distributed. Figure 3B shows the distribution of the empirically derived *R* in these studies, which confirms that the majority of the empirical distributions of the initial estimates are better described by a heavy-tailed distribution than a thin-tailed one.

We refer to the average estimate of the individuals in each group, *before* and *after* their interactions, as their collective *initial* and *revised* estimates, respectively. For each trial, we compare the absolute errors of the collective initial and revised estimates using the following two outcome metrics: (1) whether the collective revised estimate is more accurate than the collective initial estimate in groups with social interaction, and (2) the standardized (z-score) absolute error of the revised collective estimate for all groups (with or without social interaction). We use a logistic regression for the former and a linear regression for the latter.

This empirical analysis relies on the following premise: the collective initial estimate corresponds to the most decentralized influence structure ($\omega =0$), and social interactions can only increase the influence centralization ($\omega >0$). For example, even in social interactions where everybody is equally connected in terms of the communication structure, some group members may become more influential than others, by virtue of being more talkative^31^, more persuasive, or more resistant to social influence^10,17^. The key insight is the fact that the collective initial estimate (pre-social interaction) eliminates the possibility of any variation in influence and is therefore equivalent to the most decentralized network. In contrast, the collective revised estimate (post-social interaction) can be influenced disproportionately by domineering individuals, and therefore can be modeled as a centralized influence network. The same insight can be extended to modeling unstructured discussion as centralized influence and the Delphi method (and other mediated communication techniques) as relatively decentralized networks. (The interested reader can find a more careful discussion in a follow-up to this paper by Becker et al.^32^ showing the application of this modeling insight and our results here to explain why unstructured discussion will sometimes outperform numeric communication and why the outcome is sometimes reversed.)

Here, we begin by using a mixed-effect model (see SI Sect. S3.1 for specification details) to test the main hypothesis predicted by our theory, namely that the effect of social influence centralization on the performance of groups is moderated by our measure of the heavy-tailedness of the initial-estimate distribution, *R*. As shown in Fig. 3C, we find that the probability that a group improves after centralized social interaction — denoted by $\Omega$ as the outcome variable of interest — is substantially explained by *R* (z-statistic $=4.16$; $p<0.001$).

In Fig. 3D, we apply another mixed-effect model (see SI Sect. S3.1 for specification details) and find that the interaction between influence centralization and *R* significantly affects the absolute error of the revised collective estimate (*b* $= -4.89$; t-statistic $=-3.91$; $p < 0.001$). Critically, the results of this empirical analysis show that variation in *R* can completely reverse the effects of social influence centralization: when $R<0.5$, the error of the revised collective estimate is lower in decentralized influence structures; whereas when $R>0.5$, the error of the revised collective estimate is lower in centralized structures (Fig. 3D).

## Discussion

The primary contribution of this paper is to reconcile previous research about a question that is fundamental to understanding the performance of groups: how does social influence impact the accuracy of collective estimates? The critical implication of our results is that the attributes of the distribution of the initial estimates (i.e., the estimation context) moderates the effect of influence centralization. Therefore, we find no support to the hypothesis that decentralized influence structures are preferred to centralized ones *independently of the estimation context*.

Thus, the effect of network structure on the collective estimation performance should be reconceptualized under a context-dependent framework, one that considers the population of individuals performing the particular task. There is no single influence structure that is better than others in all contexts. Such a context-dependent framework can unify previously conflicting findings on crowd wisdom under a single theoretical framework and explain the effects of the influence network structure on the quality of the collective estimates. Admittedly, the estimation context is only one of several potential sources of inconsistency in previous studies. For instance, vagueness or ambiguity of some theoretical constructs, such as influence, can result in different studies of seemingly the same phenomenon measuring different things.

Although the calculation of our proposed feature of the estimation context, *R*, does not require knowledge of the truth (estimand), it does require access to a group’s set of initial estimates. However, we note that research on simple estimation tasks demonstrated that similar classes of estimation tasks tend to yield similar and reliably predictable distributions of initial estimates^20^. Thus, prescriptively, for a group that may regularly need to complete the same class of estimation tasks, it may be possible to calibrate group structure based on historical data and long-term feedback.

Furthermore, the performance of the influence structures can vary significantly as a function of the selected loss function. In this paper, our loss function is defined as the probability that the collective estimate generated by a centralized influence structure is closer to the truth than that of a decentralized structure. However, the choice of the loss function is typically application dependent. See Fig. S4 for examples of other loss functions.

Finally, we note that we only studied one class of tasks: numerical estimation with a non-negative, objective truth. Relevant research on other classes of tasks has similarly demonstrated that variation in context features, such as complexity^33–38^, fundamentally alter collective problem-solving outcomes. Also worth noting is our focus on tasks involving social influence when, in reality, we should not rule out the possibility that simply aggregating individuals’ pre-influence estimates might be an appropriate, costless solution for some situations. However, such situations fall beyond the scope of the present work and we refer the interested reader to the extensive existing literature on aggregation rules (e.g.,^39–41^).

To conclude, our theoretical and empirical analysis has demonstrated that conclusions about the role of the social influence can be inconsistent unless the estimation context is explicitly accounted for. Many research extensions are warranted from this framework. For example, unlike what is assumed in most available work, including ours, the social networks we live in are not random, nor are they imposed by external forces. Rather, these social networks emerge under the influence of endogenous social processes and gradually evolve within a potentially non-stationary context. An important avenue for future work is to investigate the consequences of correlated placement of individuals, whereby more accurate individuals are more or less likely to occupy more influential positions. We expect that as the degree of this correlation increases, the benefits or harms of centralization will become prominent and persist more consistently over a wider range of tasks and context features. A truly context-dependent view on crowd wisdom should open connections with diverse research fields and help advance an interdisciplinary understanding of the design of social systems and their information outcomes.

## Materials and methods

### Theoretical analysis of $\Omega$

We measure the probability that the collective estimate produced by a centralized influence structure, ${{\mathbf {a}}}^{n}(\omega )$, $\omega > 0$, outperforms the decentralized baseline, ${{\mathbf {a}}}^{n}(0)$. We denote this probability by $\Omega _n(\omega ,{\mathcal {F}}^{\theta }_{\mu ,\sigma }) := {\mathbb {P}}^{\theta }_{\mu ,\sigma }[|{{\mathbf {a}}}^{n}(\omega ) - \theta | < |{{\mathbf {a}}}^{n}(0) - \theta |]$. To compute $\Omega _n$ in Fig. 2, we have fixed $n=50$, $\theta = 2$, and $\omega = 1/3$. Therefore, $\Omega$ is entirely determined by the distribution of the initial estimates ($\mu$ and $\sigma$). Figure S3 replicates our simulation for a range of *n* and $\omega$ values. For distributions ${\mathcal {F}}_{\mu ,\sigma }^{\theta }$, supported over positive reals, with cumulative function ${F}_{\mu ,\sigma }^{\theta }$, we propose the following lower bound (proved in SI Sect. S2.1):

$$\begin{aligned} \Omega _n(\omega ,{\mathcal {F}}_{\mu ,\sigma }^{\theta }) \ge \displaystyle \sup _{\beta >\theta /(1-\omega )} \left\{ F^{\theta }_{\mu ,\sigma }(\beta )(1 - {F}^{\theta }_{\mu ,\sigma }(n\beta )^{n-1})\right\} . \end{aligned}$$

In SI Sect. S2.1, we show how to limit the rate of tail decay for different classes of distributions to produce a non-trivial (non-zero) lower bound as $n \rightarrow \infty$. For heavy-tailed distributions, such as Pareto, log-Laplace, and log-normal (see SI Sects. S2.1.1–S2.1.3), we identify phase-transition behaviors, whereby the proposed lower bound’s limiting value transitions from 0 to 1 or 1/2 as $\sigma$ crosses a critical value.

### Statistical tests

All statistics were two-tailed and based on mixed-effects models that included random effects by group and by study (i.e., the four human-subject laboratory experiments that we reanalyzed^9,10,14,16^) to account for the nested structure of the data. In particular, the logistic regression for Fig. 3C is:

$$\begin{aligned} y_{ij} = \frac{1}{1+\exp (b_0 + b_1 R_{j} + v_i + u_i + \epsilon _{ij})}, \end{aligned}$$

where $y_{ij}$ is a binary indicator for whether or not the *i*-th group in the *j*-th estimation context improved the accuracy of its collective estimate after social interaction; $b_0$ is the fixed intercept for the regression model; $b_1$ is the fixed coefficient for the estimation-context feature, *R*; $v_i$ is the random coefficient for the *i*-th group; $u_i$ is the random coefficient for the study that the *i*-th group belongs to; and $\epsilon _{ij}$ is a Gaussian error term. The analysis was conducted on 678 observations (groups with social influence).

The regression equation for Fig. 3D is:

$$\begin{aligned} y_{ij} = b_0 + b_1 R_{j} + b_2 I_{i} + b_3 I_{i} R_{j} + v_i + u_i + \epsilon _{ij}, \end{aligned}$$

where $y_{ij}$ is the standardized (z-score) absolute error of the revised collective estimate for the *i*-th group in the *j*-th estimation context, $R_{j}$; $b_0$ is the fixed intercept for the regression model; $b_1$ is the fixed coefficient for the estimation-context feature, *R*; $I_{i} \in \{0,1\}$ is an indicator variable of whether or not social interaction has occurred; $b_2$ is the fixed coefficient for the social influence centralization; $b_3$ is the fixed coefficient for the interaction term between the estimation-context feature, *R*, and influence centralization (shown in Fig. 3D); $v_i$ is the random coefficient for the *i*-th group; $u_i$ is the random coefficient for the study that the *i*-th group belongs to; and $\epsilon _{ij}$ is a Gaussian error term. The absolute error of the revised collective estimate has been standardized (z-scored) to compare errors across different tasks (the correct answer for different tasks can differ by orders of magnitude). The analysis was conducted on 687 observations; 582 groups with social influence (centralized) and 105 groups without social influence (decentralized). Further details of the regression analysis are provided in SI Sect. S3.1 and Table S1. Robustness checks for the regression results are presented in Tables S2 and S3.

## Supplementary Information

Supplementary Information.

## Data Availability

At https://github.com/amaatouq/task-dependence.
