# Supplementary material for: The distribution of initial estimates moderates the effect of social influence on the wisdom of the crowd
Source: Sci Rep. 2022 Oct 3;12:16546. doi: 10.1038/s41598-022-20551-7 (PMC9530231; doi:10.1038/s41598-022-20551-7)
Supplement: Supplementary file 1 — Supplementary Information. [file 41598_2022_20551_MOESM1_ESM.pdf]

Supplementary Information for: The Distribution of Initial Estimates Moderates the Effect of Social Influence on the Wisdom of Crowd

This Supplementary Information is organized in five sections. In section S1, we present network models of collective estimation that facilitate our study of the collective estimate, $\mathbf{a}^n(\omega)$, as a function of the centralization, ω, and in relation to its network structure. In section S2, we give the proof of our main proposition (lower bound on Ω_n) and relate it to the rate of tail decay for many common distributions. We study these relationships theoretically and through numerical analysis as well. In section S3, we develop empirical measures to analyze prior experiments in terms of the features of their estimation context. In section S4, we provide robustness checks. Supplementary references are listed in section S5.

Contents

S1 Network models of collective estimation **S2**

S1.1 ω: Parameterizing a class of networks by their centralization S3

S1.2 Ω: Will the estimation context benefit from centralization? S5

S2 Theoretical analysis of Ω **S7**

S2.1 The main proposition S7

S2.2 Numerical simulations S19

S3 Empirical analysis of estimation contexts in prior work **S21**

S3.1 Regression Analysis S22

S3.2 Regression Analysis Experiment-by-Experiment S23

S4 Robustness checks **S25**

S5 References **S28**

Preliminary notation. For the convenience of the reader, we collect here some notation that will be used throughout. We study collective estimation by a group of agents and use n to denote the group size. For sequences of real numbers f_n and g_n, indexed by integers n, we use the asymptotic notations $f_n \asymp g_n$ to signify that $\lim_{n\to\infty} f_n/g_n = 1$. We consider agents that are indexed by $i = 1, \dots, n$. We use bold fonts to represent random variables. We use $\mathbf{a}_{1,0}$, ..., $\mathbf{a}_{n,0}$ to denote the individual estimates in the absence of any social interactions. The

S1 Network models of collective estimation

Let θ be an unknown state of the world. Consider n agents indexed by $i = 1, \ldots, n$, each endowed with a biased and noisy signal about θ. The signals are independent and identically distributed across the agents and constitute their initial estimates of the unknown θ:

$$\mathbf{a}_{i,0} \sim \mathcal{F}^{\theta}_{\mu,\sigma}. \tag{S.1}$$

The the distribution of the initial estimates in (S.1), $\mathcal{F}^{\theta}_{\mu,\sigma}$, is parametrized by θ, μ, and σ. We think of μ as a location parameter (the center of the distribution) that biases the individual estimates against θ. This captures the level of systematic bias in the population. We think of σ as a variance-proxy/shape parameter that determines the variation and tail-fatness of $\mathcal{F}^{\theta}_{\mu,\sigma}$. In other words, σ can be interpreted as the amount of prior information a group has about the quantity and represents the level of *demonstrability* of the estimation task.

The agents interact in a group. Their group interactions can be modeled in a variety of ways leading to a group aggregate $\mathbf{a}^n(\bar{w})$ that is a convex combination of the initial estimates:

$$\mathbf{a}^n(\bar{w}) := \bar{w}^T \bar{\mathbf{a}}_0 = \sum_{i=1}^{n} w_i \mathbf{a}_{i,0}, \tag{S.2}$$

where $\bar{w}$ is an entry-wise non-negative vector satisfying $\bar{w}^T \mathbb{1} = 1$.

In general, different agents' initial estimates will receive different weights in the collective estimate. A common method of modeling group interactions is through DeGroot-style iterated averaging, which has a long history in mathematical sociology and social psychology [1]. The origins of iterated averaging models can be traced to French's seminal work on "A Formal Theory of Social Power" [2], followed up by Harary's investigation of the mathematical properties of the averaging model, including the consensus criteria, and its relations to Markov chain theory [3]. This model was further popularized by DeGroot's seminal work [4] on linear opinion pools and belief exchange dynamics. In a typical iterated averaging setup, an agent's estimate

collective estimate is denoted by $\mathbf{a}^n$; it is determined in terms of the individual estimates, in a manner that involves a centralization parameter ω: $\mathbf{a}^n(\omega) = \omega \mathbf{a}_{1,0} + (1-\omega)\frac{1}{n}\sum_{i=1}^{n} \mathbf{a}_{i,0}$. Vectors are denoted by a bar on top of their letters and we use superscript ^{T} to denote matrix transpose.

at time t is given by a weighted average of her neighboring estimates at time $t-1$:

$$\mathbf{a}_{i,t} = \sum_{j=1}^{n} W_{ij}\mathbf{a}_{j,t-1},$$

In matrix notation, we have:

$$\bar{\mathbf{a}}_t = W\bar{\mathbf{a}}_{t-1} = W^t\bar{\mathbf{a}}_0, \tag{S.3}$$

where $\bar{\mathbf{a}}_t = (\mathbf{a}_{1,t}, \mathbf{a}_{2,t}, \ldots, \mathbf{a}_{n,t})^T$ and $W = [W_{ij}]$ is the matrix of weights. We refer to matrix W as the social influence matrix. For a strongly connected social network with $W_{ii} > 0$ for all i, the Perron-Frobenius theory [5, Theorems 1.5 and 1.7] implies that W has a simple positive real eigenvalue equal to 1. Moreover, the left and right eigenspaces associated with the unit eigenvalue are both one-dimensional with the corresponding eigenvectors $\overline{w} = (w_1, \ldots, w_n)^T$ and $\mathbb{1} = (1, \ldots, 1)^T$. The magnitude of any other eigenvalue of W is strictly less than 1; therefore, we have:

$$\lim_{t\to\infty} \bar{\mathbf{a}}_t = \lim_{t\to\infty} W^t\bar{\mathbf{a}}_0 = \mathbb{1}\bar{w}^T\bar{\mathbf{a}}_0 = \mathbb{1}\mathbf{a}^n(\bar{w}), \tag{S.4}$$

which implies a consensus on the collective estimate (S.2). In several experimental settings [6, 7, 8, 9], human participants get to revise their numerical estimates a few times only, and the collective estimate is then calculated by averaging the revised estimates. Let us denote the number of communication rounds in such a scenario by τ. Using (S.3) to model the revision of the numerical estimates, we again arrive at a model that gives the collective estimate as a convex combination of the initial estimates, $\mathbf{a}^n(\bar{w}_\tau) = \bar{w}_\tau^T\bar{\mathbf{a}}_0$, where the transposed vector of weights, $\bar{w}_\tau^T$, is given by $\bar{w}_\tau^T = (1/n)\mathbb{1}^T W^\tau$.

S1.1 ω: Parameterizing a class of networks by their centralization

Motivated by our interest in comparing the collective estimation performance of centralized and decentralized networks, we focus our attention on a class of social influence network structures for which the collective estimate can be written as follows:

$$\mathbf{a}^n(\omega) = \omega\mathbf{a}_{1,0} + (1-\omega)\frac{1}{n}\sum_{i=1}^{n}\mathbf{a}_{i,0}, \tag{S.5}$$

where ω is a measure of influence centralization, with $\omega = 1$ representing a fully centralized social influence structure ($w_1 = 1$, and $w_2 = \ldots = w_n = 0$) and $\omega = 0$ corresponding to a fully decentralized social influence structure ($w_1 = \ldots = w_n = 1/n$). Indeed, with $\bar{w}$ as the centrality vector, the parameter ω corresponds to the Freeman centralization of the underlying network with social influence matrix W. By varying $\omega \in [0, 1]$, we can interpolate between the two extremes: full centralization, $\omega = 1$, and complete decentralization, $\omega = 0$. This class, although not encompassing, is ideal for addressing the central question of interest in this work. The networks in this class are such that one agent, $i = 1$, is distinguished with a higher influence and all others, $i > 1$, have an equal but lower influence. Networks in this class include cases of practical and empirical interest, such as star networks and circular lattices. All networks in Figure 1.B, in the main text, belong to this class.

Here, we consider the special cases of star and cycle networks with bidirectional edges, and equal weights on all edges that are incoming to the same node. Their respective social influence matrices and associated left eigenvectors are given by:

$$W_\star = \begin{bmatrix} 1/n & 1/n & 1/n & \cdots & 1/n \\ 1/2 & 1/2 & 0 & \cdots & 0 \\ 1/2 & 0 & 1/2 & 0\cdots & 0 \\ \vdots & \vdots & \ddots & \vdots & \vdots \\ & & & & 0 \\ 1/2 & 0 & \cdots & 0 & 1/2 \end{bmatrix}, \quad W_o = \begin{bmatrix} 1/3 & 1/3 & 0 & \cdots 0 & 1/3 \\ 1/3 & 1/3 & 1/3 & 0 \ldots & 0 \\ 0 & \ddots & \ddots & & 0 \\ \vdots & & & & \\ 0 & \ddots 0 & 1/3 & 1/3 & 1/3 \\ 1/3 & \ldots & 0 & 1/3 & 1/3 \end{bmatrix},$$

$$\bar{w}_\star = (n/(3n-2), 2/(3n-2), \ldots, 2/(3n-2))^T, \;\; \bar{w}_o = (1/n, 1/n, \ldots, 1/n)^T.$$

Subsequently, the network centralization parameter ω in (S.5) for the star and cycle networks are given by $\omega_\star = (n-2)/(3n-2)$ and $\omega_o = 0$. Note that as $n \to \infty$, $\omega_\star \to 1/3$. This, together with the fact that experimental studies have used the star topology to test collective estimation in centralized structures [7], motivates our choice of $\omega = 1/3$ in numerical simulations and empirical analysis. In section S4, we show that our results are robust to this choice of ω.

Although our main proposition in section S2.1 gives a lower bound that is valid for any n, most of the subsequent analysis concerns the limiting behavior of the collective estimates as $n \to \infty$. Our results extend to networks with a finite (non-increasing) collection of influ-

ential agents. To accommodate such cases, one would replace $\mathbf{a}_{1,0}$ in (S.5) with the (possibly weighted) average of the initial estimates of the k influential agents, for some fixed constant k. One can similarly consider generalizations where the remaining, non-influential agents have unequal — but all vanishing — weights (going to zero as $n \to \infty$).

S1.2 Ω: Will the estimation context benefit from centralization?

We consider a case where agents are randomly placed in the social influence network. This is typical of many experimental setups [8, 7, 6]. Let $\mathbb{E}^{\theta}_{\mu,\sigma}$ be the expectation with respect to the random draws of the n i.i.d. initial estimates, $\mathbf{a}_{1,0}, \ldots, \mathbf{a}_{n,0} \sim \mathcal{F}^{\theta}_{\mu,\sigma}$, and let $\mathbb{P}^{\theta}_{\mu,\sigma}$ be the corresponding probability measure. The expected mean square root error, mean absolute error, and mean squared error of the collective estimate are given by:

$$\mathrm{MSRE}_n(\bar{w}, \mathcal{F}^{\theta}_{\mu,\sigma}) := \mathbb{E}^{\theta}_{\mu,\sigma}\left[|\mathbf{a}^n(\bar{w}) - \theta|^{1/2}\right],$$
$$\mathrm{MAE}_n(\bar{w}, \mathcal{F}^{\theta}_{\mu,\sigma}) := \mathbb{E}^{\theta}_{\mu,\sigma}\left[|\mathbf{a}^n(\bar{w}) - \theta|\right],$$
$$\mathrm{MSE}_n(\bar{w}, \mathcal{F}^{\theta}_{\mu,\sigma}) := \mathbb{E}^{\theta}_{\mu,\sigma}\left[(\mathbf{a}^n(\bar{w}) - \theta)^2\right].$$

In order to investigate the interaction between the network structure and the distribution of the initial estimates, (i.e. the *estimation context*: a population of agents performing a particular estimation task), we propose the following measure of how the collective estimate $\mathbf{a}^n(\bar{w}) = \bar{w}^T\bar{\mathbf{a}}_0$ performs against a fully decentralized aggregate $\mathbf{a}^n(0)$:

$$\Omega_n(\overline{w}, \mathcal{F}^{\theta}_{\mu,\sigma}) := \mathbb{P}^{\theta}_{\mu,\sigma}[|\mathbf{a}^n(\bar{w}) - \theta| < |\mathbf{a}^n(0) - \theta|],$$

where $\overline{w}$ is the centrality vector in (S.4). Restricting attention to the class of networks in subsection S1.1, with $\mathbf{a}^n(\omega) = \omega\mathbf{a}_{1,0} + (1-\omega)\frac{1}{n}\sum_{i=1}^{n}\mathbf{a}_{i,0}$, we can write:

$$\Omega_n(\omega, \mathcal{F}^{\theta}_{\mu,\sigma}) = \mathbb{P}^{\theta}_{\mu,\sigma}[|\mathbf{a}^n(\omega) - \theta| < |\mathbf{a}^n(0) - \theta|].$$

This measure, $\Omega_n(\omega, \mathcal{F}^{\theta}_{\mu,\sigma})$, corresponds to the probability that a network with social influence centralization $\omega > 0$ outperforms a decentralized network with $\omega = 0$, in absolute error perfor-

mance. Similarly, for other performance measures, we can write:

$$\begin{aligned} \text{MSRE}_n(\omega, \mathcal{F}^{\theta}_{\mu,\sigma}) &:= \mathbb{E}^{\theta}_{\mu,\sigma}\left[|\mathbf{a}^n(\omega) - \theta|^{1/2}\right], \\ \text{MAE}_n(\omega, \mathcal{F}^{\theta}_{\mu,\sigma}) &:= \mathbb{E}^{\theta}_{\mu,\sigma}\left[|\mathbf{a}^n(\omega) - \theta|\right], \\ \text{MSE}_n(\omega, \mathcal{F}^{\theta}_{\mu,\sigma}) &:= \mathbb{E}^{\theta}_{\mu,\sigma}\left[(\mathbf{a}^n(\omega) - \theta)^2\right]. \end{aligned} \tag{S.6}$$

Our focus in section S2 is on Ω_n, as the probability of the outcome of interest, to understand if and when the estimation context will benefit from centralization. In section S2, we present a theoretical and numerical analysis of the properties of Ω_n. In particular, we show how the behavior of Ω_n varies with the estimation context, i.e. the distribution of the initial estimates. In section S2.1, we present a theoretical lower bound on $\Omega_n(\omega, \mathcal{F}^{\theta}_{\mu,\sigma})$ and analyze its behavior for various classes of distributions, $\mathcal{F}^{\theta}_{\mu,\sigma}$. In section S2.2, we supplement these findings by numerical analysis and simulations. We present our numerical results in the main text for $\omega = 1/3$ and $n = 50$. In section S4 we show that our results are robust to our choices of ω and n. Of note, Ω_n is concerned only with the probability of the following event: the collective estimate generated by the agents interacting in a centralized influence structure will be closer to the truth than the collective estimate generated by the agents in a decentralized structure. This is not the same as comparing the expected loss or error magnitudes. In section S4, Figure S4, we show the results for various loss function choices.

In section S3, we propose a feature of the estimation context that can be empirically measured to predict when social influence improves the collective estimation accuracy in prior empirical studies. The proposed feature, R, is based on how heavy-tailed the distribution of the initial estimates is. It is defined as the relative fit (measured in log likelihood) of a log-normal versus a normal distribution to the empirical distribution of the initial estimates. In particular, in experimental conditions with no social interactions, an external observers polls each of the participants for their opinions. Therefore, in the absence of social influence, the aggregate is given by $\mathbf{a}^n(0) = (1/n)\sum_{i=1}^{n} \mathbf{a}_{i,0}$, which is equivalent to a fully decentralized influence structure. On the other hand, in the presence of social influence, the participants revise their estimates as a result of their social interactions, thus leading to an aggregate that is a weighted average of the initial estimate, $\mathbf{a}^n(\bar{w}) = \sum_{i=1}^{n} w_i \mathbf{a}_{i,0}$. Hence, social interaction leads to a collective estimate that is less decentralized. In our model, we capture this case by $\mathbf{a}^n(\omega)$ for some $\omega > 0$. Our empirical results show that R can significantly predict when the latter is more accurate than $\mathbf{a}^n(0)$.

S2 Theoretical analysis of Ω

In this section, we first propose a lower bound on $\Omega_n(\omega, \mathcal{F}^{\theta}_{\mu,\sigma})$ in subsection S2.1, followed by analyses of its behavior for different distributions in five sub-subsections: these are the Pareto, S2.1.1, log-Laplace, S2.1.2, log-normal, S2.1.3, other heavy-tailed, S2.1.4, and thin-tailed distributions, S2.1.5. In subsection S2.2, we describe the procedure for direct numerical simulations of Ω for these various classes and offer additional numerical insights.

S2.1 The main proposition

Motivated by empirical literature that pose estimation questions to human participants, we focus on $0 < \theta$ and distributions $\mathcal{F}^{\theta}_{\mu,\sigma}$ with support over positive reals. Fix $0 < \theta$, $0 < \omega < 1$, $\theta/(1-\omega) < \beta$, and consider the event $\mathcal{E}_1 = \{\mathbf{a}_{1,0} < \beta\}$. Note that for many distributions we can make $\mathbb{P}^{\theta}_{\mu,\sigma}(\mathcal{E}_1)$ arbitrarily close to one by taking β large enough. Next consider the event $\mathcal{E}_n = \{\mathbf{a}^n(0) > \beta + \mathbf{a}_{1,0}/n\}$. Note that $\mathcal{E}_n$ implies $\mathbf{a}^n(0) > \beta$; furthermore $\mathcal{E}_1$ and $\mathcal{E}_n$ are independent events. On the other hand, conditioned on the events $\mathcal{E}_1$ and $\mathcal{E}_n$, we have:

$$\begin{aligned}
|\mathbf{a}^n(\omega) - \theta| &= |\omega \mathbf{a}_{1,0} + (1-\omega)\mathbf{a}^n(0) - \theta| < \omega \mathbf{a}_{1,0} + |(1-\omega)\mathbf{a}^n(0) - \theta| \\
&< \omega\beta + |(1-\omega)\mathbf{a}^n(0) - \theta| \\
&= \omega\beta + (1-\omega)\mathbf{a}^n(0) - \theta < \omega\mathbf{a}^n(0) + (1-\omega)\mathbf{a}^n(0) - \theta = |\mathbf{a}^n(0) - \theta|,
\end{aligned}$$

where in the second line we have used $\beta > \mathbf{a}_{1,0}$, and in the third line we have used $\mathbf{a}^n(0) > \beta$ and $(1-\omega)\mathbf{a}^n(0) > (1-\omega)\beta > \theta$. Hence, conditioned on $\mathcal{E}_1 \cap \mathcal{E}_n$, we have $|\mathbf{a}^n(\omega) - \theta| < |\mathbf{a}^n(0) - \theta|$, i.e., centralized networks outperform decentralized ones. We can thus bound $\Omega_n(\omega, \mathcal{F}^{\theta}_{\mu,\sigma})$, the probability that a social influence network with centralization ω outperforms a decentralized one ($\omega = 0$) in absolute error measure:

$$\Omega_n(\omega, \mathcal{F}^{\theta}_{\mu,\sigma}) \geq \mathbb{P}^{\theta}_{\mu,\sigma}[\mathcal{E}_1 \cap \mathcal{E}_n] = \mathbb{P}^{\theta}_{\mu,\sigma}[\mathcal{E}_1]\mathbb{P}^{\theta}_{\mu,\sigma}[\mathcal{E}_n]. \tag{S.7}$$

Denoting the cumulative distribution $F^{\theta}_{\mu,\sigma}(x) := \mathbb{P}^{\theta}_{\mu,\sigma}[\mathbf{a}_{1,0} \leq x]$ and the tail probability $\bar{F}^{\theta}_{\mu,\sigma}(x) := \mathbb{P}^{\theta}_{\mu,\sigma}[\mathbf{a}_{1,0} > x]$, we can write $\mathbb{P}^{\theta}_{\mu,\sigma}[\mathcal{E}_1] = \mathbb{P}^{\theta}_{\mu,\sigma}[\mathbf{a}_{1,0} \leq \beta] = F^{\theta}_{\mu,\sigma}(\beta)$, and

$$\mathbb{P}^{\theta}_{\mu,\sigma}[\mathcal{E}_n] = \mathbb{P}^{\theta}_{\mu,\sigma}[\,\textstyle\sum_2^n \mathbf{a}_{i,0} > n\beta\,] \geq \mathbb{P}^{\theta}_{\mu,\sigma}[\,\max\limits_{2,\dots,n} \mathbf{a}_{i,0} > n\beta\,] = 1 - F^{\theta}_{\mu,\sigma}(n\beta)^{n-1}. \tag{S.8}$$

Using $\mathbb{P}^{\theta}_{\mu,\sigma}[\mathcal{E}_1]\mathbb{P}^{\theta}_{\mu,\sigma}[\mathcal{E}_n] \geq F^{\theta}_{\mu,\sigma}(\beta)(1 - F^{\theta}_{\mu,\sigma}(n\beta)^{n-1})$, together with (S.7), we arrive at our main proposition:

$$\textbf{Proposition}.\ \Omega_n(\omega, \mathcal{F}^{\theta}_{\mu,\sigma}) \geq \sup_{\beta > \theta/(1-\omega)} \left\{ F^{\theta}_{\mu,\sigma}(\beta)(1 - F^{\theta}_{\mu,\sigma}(n\beta)^{n-1}) \right\}.$$

To proceed, let us denote the lower bound:

$$\underline{\Omega}_n(\omega, \mathcal{F}^{\theta}_{\mu,\sigma}) := \sup_{\beta > \theta/(1-\omega)} \left\{ F^{\theta}_{\mu,\sigma}(\beta)(1 - F^{\theta}_{\mu,\sigma}(n\beta)^{n-1}) \right\}. \tag{S.9}$$

Some observations are now in order regarding the behavior of the lower bound, $\underline{\Omega}_n(\omega, \mathcal{F}^{\theta}_{\mu,\sigma})$. First note that $\underline{\Omega}_n(\omega, \mathcal{F}^{\theta}_{\mu,\sigma})$ is decreasing in θ and ω. Secondly, the asymptotic behavior of $\underline{\Omega}_n(\omega, \mathcal{F}^{\theta}_{\mu,\sigma})$, as $n \to \infty$, is determined by $F^{\theta}_{\mu,\sigma}(n\beta)^{n-1}$. Note that $F^{\theta}_{\mu,\sigma}(n\beta) \leq 1$ and $F^{\theta}_{\mu,\sigma}(\beta) \to 1$ as $n \to \infty$ for any $\beta > 0$. Therefore, if $F^{\theta}_{\mu,\sigma}(n\beta) \to 1$ at a slow enough rate such that $F^{\theta}_{\mu,\sigma}(n\beta)^{n-1}$ is bounded away from one for all n, then $F^{\theta}_{\mu,\sigma}(\beta)(1 - F^{\theta}_{\mu,\sigma}(n\beta)^{n-1})$ is bounded away from zero as $n \to \infty$. Subsequently, for various classes of distributions with slow enough tail decay, we can establish nontrivial lower bounds $\underline{\Omega}_n(\omega, \mathcal{F}^{\theta}_{\mu,\sigma}) > 0$.

In subsections S2.1.1 to S2.1.3, we give conditions on the estimation context, i.e., the distribution of the initial estimates (parameterized by μ, σ, and θ), of well-known heavy-tailed distributions such that $\underline{\Omega}_n, \Omega_n \to 1$ as $n \to \infty$. In subsection S2.1.4, we discuss the general properties of heavy-tailed distributions that make them relevant to our proposition. Finally, in subsection S2.1.5 we present countervailing arguments for thin-tailed distributions.

S2.1.1 Pareto (power-law)

Pareto or power-law distributions are archetypal, heavy-tailed distributions characterized by their polynomial tail decay. Consider a Pareto distribution with location parameter θe^{μ} and

shape parameter σ, defined as follows:

$$\bar{F}^{\theta}_{\mu,\sigma}(x) = (\theta e^{\mu}/x)^{1/\sigma}, \text{ for } x \geq \theta e^{\mu}. \tag{S.10}$$

For Pareto distributions we have:

$$\begin{aligned} F^{\theta}_{\mu,\sigma}(\beta)(1 - F^{\theta}_{\mu,\sigma}(n\beta)^{n-1}) &= \left(1 - \left(\frac{\theta e^{\mu}}{\beta}\right)^{1/\sigma}\right)\left(1 - \left(1 - \left(\frac{\theta e^{\mu}}{n\beta}\right)^{1/\sigma}\right)^{n-1}\right) \\ &\asymp \left(1 - \left(\frac{\theta e^{\mu}}{\beta}\right)^{1/\sigma}\right)\left(1 - e^{-n(\theta e^{\mu}/n\beta)^{1/\sigma}}\right), \end{aligned} \tag{S.11}$$

where we used the $n \to \infty$ asymptotic equality

$$e^{-n(\theta e^{\mu}/n\beta)^{1/\sigma}} \asymp \left(1 - \left(\frac{\theta e^{\mu}}{n\beta}\right)^{1/\sigma}\right)^{n-1}. \tag{S.12}$$

We now consider the three distinct $n \to \infty$ limiting behavior that arise for $\sigma > 1$, $\sigma = 1$, and $\sigma < 1$:

- For $\sigma > 1$, as $n \to \infty$ we get

  $$\underline{\Omega}_n(\omega, \mathcal{F}^{\theta}_{\mu,\sigma}) \geq 1 - (\theta e^{\mu}/\beta)^{1/\sigma} \text{ for all } \beta > \theta/(1-\omega),$$

  and letting $\beta \to \infty$ we conclude that

  $$\Omega_n(\omega, \mathcal{F}^{\theta}_{\mu,\sigma}) \asymp \underline{\Omega}_n(\omega, \mathcal{F}^{\theta}_{\mu,\sigma}) \asymp 1, \text{for } \sigma > 1, \text{ and any } 0 < \theta, 0 < \omega < 1, \text{ and real } \mu.$$

- Replacing $\sigma = 1$ in (S.11), the lower bound $\underline{\Omega}_n(\omega, \mathcal{F}^{\theta}_{\mu,1})$ can be calculated as follow:

  $$\begin{aligned} &\sup_{\beta > \theta/(1-\omega)} \left\{1 - \frac{\theta e^{\mu}}{\beta} - e^{-\theta e^{\mu}/\beta} + \frac{\theta e^{\mu}}{\beta} e^{-\theta e^{\mu}/\beta}\right\} \\ &= \begin{cases} W_0(e^2) + 1/W_0(e^2) - 2 \approx 0.199, & \text{if } \mu \geq \log\left(\frac{2 - W_0(e^2)}{1-\omega}\right), \\ 1 - e^{\mu}(1-\omega) - e^{-e^{\mu}(1-\omega)} + e^{\mu}(1-\omega)e^{-e^{\mu}(1-\omega)}, & \text{if } \mu < \log\left(\frac{2 - W_0(e^2)}{1-\omega}\right). \end{cases} \end{aligned}$$

To see why, denote $x = \theta e^{\mu}/\beta$. The maximum of $1-x-e^{-x}+xe^{-x}$ occurs at $x^\star$ satisfying $-1+2e^{-x^\star} - x^\star e^{-x^\star} = 0$. The latter has a unique solution over the positive reals given by $x^\star = 2 - W_0(e^2) \approx 0.443$, where W_0 is the principal branch Lambert W function, uniquely satisfying $W_0(e^2)e^{W_0(e^2)} = e^2$ on positive reals. Replacing $x = 2 - W_0(e^2)$ in $1 - x - e^{-x} + xe^{-x}$ gives the maximum value $W_0(e^2) + 1/W_0(e^2) - 2$ realized at $\beta = \theta e^{\mu}/(2 - W_0(e^2))$ if $e^{\mu} \geq (2 - W_0(e^2))/(1-\omega)$. If $e^{\mu} < (2 - W_0(e^2))/(1-\omega)$, then the supremum is achieved with $\beta = \theta/(1-\omega)$ at a value that is strictly less than $W_0(e^2) + 1/W_0(e^2) - 2 \approx 0.199$.

- For $\sigma < 1$, as $n \to \infty$ we get

$$\left(1 - \left(\frac{\theta e^{\mu}}{\beta}\right)^{1/\sigma}\right)\left(1 - e^{-n(\theta e^{\mu}/n\beta)^{1/\sigma}}\right) \to 0.$$

  for any β.

We thus obtain the following asymptotic characterization of the lower bound for Pareto distributions (indicating a phase transition at $\sigma = 1$):

$$\underline{\Omega}_n(\omega, \mathcal{F}^{\theta}_{\mu,\sigma}) \asymp \begin{cases} 0, & \text{if } \sigma < 1, \\ (1 - e^{\mu}(1-\omega))(1 - e^{-e^{\mu}(1-\omega)}), & \text{if } \sigma = 1 \text{ and } \mu < \log\left(\frac{2-W_0(e^2)}{1-\omega}\right), \\ W_0(e^2) + 1/W_0(e^2) - 2 \approx 0.199, & \text{if } \sigma = 1 \text{ and } \mu \geq \log\left(\frac{2-W_0(e^2)}{1-\omega}\right), \\ 1, & \text{if } \sigma > 1. \end{cases} \tag{S.13}$$

In Figure S1.A, top, we have plotted (S.13) for $\omega = 1/3$, $\theta = 2$, and $n = 50$. Comparing with the direct numerical simulation in Figure S1.A, bottom, shows how the bound gets tighter for large σ.

S2.1.2 Log-Laplace

Jayles et al. [10] point out that log-Laplace provides a better fit to the empirically measured distribution of the initial estimates, compared to log-Cauchy [11], or log-normal. Here, we analyze the asymptotic behavior of the proposed lower bound as $n \to \infty$, when the initial estimates are distributed according to a log-Laplace distribution with parameters $\log\theta + \mu$ and

σ:

$$F^{\theta}_{\mu,\sigma}(x) = \begin{cases} \frac{1}{2}\exp\left(\frac{\log(x/\theta)-\mu}{\sigma}\right), & \text{if } \log(x/\theta) < \mu, \\ 1 - \frac{1}{2}\exp\left(\frac{\mu-\log(x/\theta)}{\sigma}\right), & \text{if } \log(x/\theta) \geq \mu. \end{cases} \tag{S.14}$$

For n large enough, we have $\log(n\beta/\theta) \geq \mu$, and using the same asymptotics as in (S.12) we get:

$$\begin{aligned} F^{\theta}_{\mu,\sigma}(n\beta)^{n-1} &= \left(1 - \frac{1}{2}\exp\left(\frac{\mu - \log(n\beta/\theta)}{\sigma}\right)\right)^{n-1} \\ &= \left(1 - \left(\frac{1}{n}\right)^{1/\sigma}\exp\left(\frac{\mu + \log\theta - \log\beta}{\sigma} - \log(2)\right)\right)^{n-1} \\ &\asymp \exp\left(-n\left(\frac{1}{n}\right)^{1/\sigma}\exp\left(\frac{\mu + \log\theta - \log\beta}{\sigma} - \log(2)\right)\right) \\ &\asymp \begin{cases} 1, & \text{if } \sigma < 1, \\ \exp\left(\frac{-e^{\mu}\theta}{2\beta}\right), & \text{if } \sigma = 1, \\ 0, & \text{if } \sigma > 1. \end{cases} \end{aligned}$$

Subsequently, for $\sigma < 1$ we have $F^{\theta}_{\mu,\sigma}(\beta)(1 - F^{\theta}_{\mu,\sigma}(n\beta)^{n-1}) \asymp 0$, for any β. On the other hand, for $\sigma > 1$ we have $F^{\theta}_{\mu,\sigma}(\beta)(1 - F^{\theta}_{\mu,\sigma}(n\beta)^{n-1}) \asymp F^{\theta}_{\mu,\sigma}(\beta)$, which is increasing in β and goes to one as β increases to ∞. Hence,

$$\underline{\Omega}_n(\omega, \mathcal{F}^{\theta}_{\mu,\sigma}) = \sup_{\beta > \theta/(1-\omega)} \left\{F^{\theta}_{\mu,\sigma}(\beta)(1 - F^{\theta}_{\mu,\sigma}(n\beta)^{n-1})\right\} \asymp 1, \text{ for } \sigma > 1.$$

Finally, for $\sigma = 1$, we have:

$$F^{\theta}_{\mu,1}(\beta)(1 - F^{\theta}_{\mu,1}(n\beta)^{n-1}) \asymp \begin{cases} \left(1 - \exp\left(\frac{-e^{\mu}\theta}{2\beta}\right)\right)\frac{\beta}{2\theta e^{\mu}}, & \text{if } \frac{\beta}{e^{\mu}\theta} < 1, \\ \left(1 - \exp\left(\frac{-e^{\mu}\theta}{2\beta}\right)\right)\left(1 - \frac{e^{\mu}\theta}{2\beta}\right), & \text{if } \frac{\beta}{e^{\mu}\theta} \geq 1. \end{cases}$$

Optimizing β gives:

$$\underline{\Omega}_n(\omega, \mathcal{F}^\theta_{\mu,1}) \asymp \begin{cases} \frac{1-1/\sqrt{e}}{2} \approx 0.1967, & \text{if } \mu \geq -\log(1-\omega), \\ \left(1 - e^{-e^\mu(1-\omega)/2}\right)(1 - e^\mu(1-\omega)/2) < \frac{1-1/\sqrt{e}}{2}, & \text{if } \mu < -\log(1-\omega). \end{cases}$$

We summarize the above results in the following asymptotic characterization of the lower bound for log-Laplace distributions, with a phase transition at $\sigma = 1$:

$$\underline{\Omega}_n(\omega, \mathcal{F}^\theta_{\mu,\sigma}) \asymp \begin{cases} 0, & \text{if } \sigma < 1, \\ \left(1 - e^{-e^\mu(1-\omega)/2}\right)(1 - e^\mu(1-\omega)/2)\,, & \text{if } \sigma = 1 \text{ and } \mu < -\log(1-\omega), \\ \frac{1-1/\sqrt{e}}{2} \approx 0.1967, & \text{if } \sigma = 1 \text{ and } \mu \geq -\log(1-\omega), \\ 1, & \text{if } \sigma > 1. \end{cases} \tag{S.15}$$

In Figure S1.B, top, we have plotted (S.15) for $\omega = 1/3$, $\theta = 2$, and $n = 50$. Comparing with the direct numerical simulation in Figure S1.B, bottom, shows how the bound gets tighter for large σ.

S2.1.3 Log-normal

Several empirical studies report a log-normal distribution for the initial estimates [7, 8, 12]. Here, we analyze the case where the initial estimates are distributed according to a log-normal distribution with parameters $\log\theta + \mu$ and σ:

$$F^\theta_{\mu,\sigma}(x) = \Phi\left(\frac{\log(x/\theta) - \mu}{\sigma}\right), x > 0, \tag{S.16}$$

where Φ is the standard normal distribution. We next apply the following control over the Gaussian tail:

$$1 - \frac{1}{\sqrt{2\pi}t} e^{-t^2/2} \leq \Phi(t) \leq 1 - \frac{t}{\sqrt{2\pi}(t^2+1)} e^{-t^2/2}$$

to obtain:

$$\left(1-\frac{\sigma}{\sqrt{2\pi}(\log(n\beta/\theta)-\mu)}\exp\left(-\frac{(\log(n\beta/\theta)-\mu)^2}{2\sigma^2}\right)\right)^{n-1} \leq F^{\theta}_{\mu,\sigma}(n\beta)^{n-1}$$
$$\leq \left(1-\frac{(\log(n\beta/\theta)-\mu)\sigma}{\sqrt{2\pi}((\log(n\beta/\theta)-\mu)^2+\sigma^2)}\exp\left(-\frac{(\log(n\beta/\theta)-\mu)^2}{2\sigma^2}\right)\right)^{n-1}. \tag{S.17}$$

We next choose $\sigma=\sigma_n$, with β and θ, fixed such that $(\log(n\beta/\theta)-\mu)/\sigma_n \to \infty$ as $n\to\infty$. Taking the limit $n\to\infty$, with $\sigma=\sigma_n$ in (S.17), we get:

$$F^{\theta}_{\mu,\sigma_n}(n\beta)^{n-1} \tag{S.18}$$
$$\asymp \exp\left(-\frac{n\sigma_n}{\sqrt{2\pi}\log n}\exp\left(-\frac{(\log(n\beta/\theta)-\mu)^2}{2\sigma_n^2}\right)\right)$$
$$= \exp\left(-\frac{1}{\sqrt{2\pi}\log n}\exp\left(\log n+\log\sigma_n-\frac{(\log(n\beta/\theta)-\mu)^2}{2\sigma_n^2}\right)\right)$$

Focusing on the second exponent, we obtain :

$$f_n := \log n+\log\sigma_n-\frac{(\log(n\beta/\theta)-\mu)^2}{2\sigma_n^2} \tag{S.19}$$
$$= \log n+\log\sigma_n-\frac{\log(n\beta/\theta)^2}{2\sigma_n^2}-\frac{\mu^2}{2\sigma_n^2}+\frac{\mu\log(n\beta/\theta)}{\sigma_n^2}$$
$$= \log n+\log\sigma_n-\frac{(\log n)^2}{2\sigma_n^2}-\frac{\log(\beta/\theta)\log n}{\sigma_n^2}-\frac{\log(\beta/\theta)^2}{2\sigma_n^2}-\frac{\mu^2}{2\sigma_n^2}+\frac{\mu\log(n\beta/\theta)}{\sigma_n^2}.$$

Setting $\sigma_n=\sqrt{\log n/k_n}$ in (S.19) yields:

$$f_n = (1-k_n/2)\log n+(1/2)\log\log n+(\mu-\log(\beta/\theta))\,k_n-(1/2)\log k_n$$
$$+\left(-(1/2)\log(\beta/\theta)^2-\mu^2/2+\mu\log(\beta/\theta)\right)\frac{k_n}{\log n}$$
$$\asymp \begin{cases} \frac{(2-k_n)}{2}\log n, & \text{if } k_n<2,\\ (1/2)\log\log n, & \text{if } k_n=2,\\ \frac{(2-k_n)}{2}\log n, & \text{if } k_n>2.\end{cases}$$

Finally, substituting in (S.18), we get:

$$F^{\theta}_{\mu,\sigma_n}(n\beta)^{n-1} \asymp \exp\Big(-e^{f_n}/(\sqrt{2\pi}\log n)\Big) \asymp \begin{cases} 0, & \text{if } \sigma_n > \sqrt{\log n/2}, \\ 1, & \text{if } \sigma_n \le \sqrt{\log n/2}. \end{cases}$$

The latter together with the fact that $F^{\theta}_{\mu,\sigma_n}(\beta) = \Phi\left((\log(\beta/\theta) - \mu)/\sigma_n\right) \asymp \Phi(0) = 1/2$, for $\sigma_n > \sqrt{\log n/2}$, leads to the following asymptotic characterization for log-normal distributions (indicating a phase transition at $\sigma_n = \sqrt{\log n/2}$):

$$F^{\theta}_{\mu,\sigma_n}(\beta)(1 - F^{\theta}_{\mu,\sigma_n}(n\beta)^{n-1}) \asymp \begin{cases} 0, & \text{if } \sigma_n \le \sqrt{\log n/2}, \\ 1/2, & \text{if } \sigma_n > \sqrt{\log n/2}, \end{cases}$$

which is true for all β, and in particular yields:

$$\begin{aligned} \underline{\Omega}_n(\omega, \mathcal{F}^{\theta}_{\mu,\sigma}) &= \sup_{\beta > \theta/(1-\omega)} \left\{F^{\theta}_{\mu,\sigma}(\beta)(1 - F^{\theta}_{\mu,\sigma}(n\beta)^{n-1})\right\} \\ &\asymp \begin{cases} 0, & \text{if } \sigma_n \le \sqrt{\log n/2}, \\ 1/2, & \text{if } \sigma_n > \sqrt{\log n/2}. \end{cases} \end{aligned} \tag{S.20}$$

In Figure S1.C, top, we have plotted (S.20) for $\omega = 1/3$, $\theta = 2$, and $n = 50$. Comparing with the result of the direct numerical simulation, Figure S1.C, bottom, shows how the bound gets tighter for larger σ.

S2.1.4 Other heavy-tailed distributions

Many empirical studies [8, 11, 12, 13, 14] point out a heavy-tailed distribution for the numerical estimates (with a few estimates that fall on a fat right tail). Following the proof of the main proposition, we pointed out that for heavy-tailed distributions where $\bar{F}^{\theta}_{\mu,\sigma}(n\beta)$ decreases slowly, we can provide non-trivial lower bounds on Ω_n that remain bounded away from zero, $\underline{\Omega}_n(\omega, \mathcal{F}^{\theta}_{\mu,\sigma}) > 0$, even as $n \to \infty$. In fact, if $\bar{F}^{\theta}_{\mu,\sigma}(n\beta)$ decreases at a rate that is slower than $1/n$, i.e., $n\bar{F}^{\theta}_{\mu,\sigma}(n\beta) \to \infty$, then $F^{\theta}_{\mu,\sigma}(n\beta)^{n-1} \to 0$ as $n \to \infty$. For such slowly decaying tails, the supremum in (S.9) is achieved as $\beta \to \infty$, and we can guarantee that $\underline{\Omega}_n \asymp \Omega_n \asymp 1$; hence, the proposed lower bound is asymptotically tight.

Here, we identify a second way, in which, our proposed lower bound is tighter for heavy-tailed distributions. To this end, let us revisit (S.8) — a critical step in deriving the proposed lower bound:

$$\mathbb{P}^{\theta}_{\mu,\sigma}[\mathcal{E}_n] = \mathbb{P}^{\theta}_{\mu,\sigma}[\,\sum_{i=2}^{n} \mathbf{a}_{i,0} > n\beta\,] > \mathbb{P}^{\theta}_{\mu,\sigma}[\,\max_{2,\ldots,n} \mathbf{a}_{i,0} > n\beta\,].$$

This inequality is at the heart of the so-called "catastrophe principle" [15, Chapter 3] that applies to many heavy-tailed distributions. Intuitively, this principle entails that when one observes a larger than expected average value for a collection of heavy-tailed random variables, then this observation is most likely explained by the existence of a very large sample in the collection, i.e. a "catastrophe". On the other hand, the countervailing explanation in the case of light-tailed random variables is that "most" of the samples in the collection happen to be larger than expected. Formally, the distribution $\mathcal{F}^{\theta}_{\mu,\sigma}$ of the initial estimates is said to satisfy the catastrophe principle [15, Definition 3.1], if for any n:

$$\lim_{t\to\infty} \frac{\mathbb{P}^{\theta}_{\mu,\sigma}[\,\max_{1,\ldots,n} \mathbf{a}_{i,0} > t\,]}{\mathbb{P}^{\theta}_{\mu,\sigma}[\,\sum_{i=1}^{n} \mathbf{a}_{i,0} > t\,]} = 1.$$

The preceding condition is equivalent to having:

$$\lim_{t\to\infty} \frac{n\mathbb{P}^{\theta}_{\mu,\sigma}[\mathbf{a}_{1,0} > t]}{\mathbb{P}^{\theta}_{\mu,\sigma}[\sum_{i=1}^{n} \mathbf{a}_{i,0} > t]} = 1, \text{ for all } n \geq 2.$$

The latter is the defining property for the subexponential family of distributions, which include many common classes of heavy-tailed distributions such as those considered in subsections S2.1.1 to S2.1.3. Setting $t = n\beta$ and letting $n \to \infty$, we obtain that if $\mathcal{F}^{\theta}_{\mu,\sigma}$ is a member of the subexponential family, then

$$\mathbb{P}^{\theta}_{\mu,\sigma}[\mathcal{E}_n] = \mathbb{P}^{\theta}_{\mu,\sigma}[\,\sum_{i=2}^{n} \mathbf{a}_{i,0} > n\beta\,] \asymp \mathbb{P}^{\theta}_{\mu,\sigma}[\,\max_{2,\ldots,n} \mathbf{a}_{i,0} > n\beta\,].$$

Hence, for such distributions belonging to the subexponential family our proposed lower bound is asymptotically tight in as much as $\mathbb{P}^{\theta}_{\mu,\sigma}[\mathcal{E}_1 \cap \mathcal{E}_n] \asymp F^{\theta}_{\mu,\sigma}(\beta)(1 - F^{\theta}_{\mu,\sigma}(n\beta)^{n-1})$, and the only way in which our lower bound may be loose is through (S.7), i.e. if $\Omega_n(\omega, \mathcal{F}^{\theta}_{\mu,\sigma}) > \mathbb{P}^{\theta}_{\mu,\sigma}[\mathcal{E}_1 \cap \mathcal{E}_n]$ for all $\beta > \theta/(1-\omega)$ as $n \to \infty$.

It is worth noting that many light-tailed distributions portray an opposite picture, referred to as "conspiracy principle" in [15, Definition 3.2]; formally defined as follows:

$$\lim_{t\to\infty} \frac{\mathbb{P}^{\theta}_{\mu,\sigma}[\max_{1,\ldots,n} \mathbf{a}_{i,0} > t\,]}{\mathbb{P}^{\theta}_{\mu,\sigma}[\sum_{i=1}^{n} \mathbf{a}_{i,0} > t]} = 0, \text{ for all } n \geq 2.$$

As an example, suppose that the initial estimates are exponentially distributed with mean θe^{μ} and the following tail probability:

$$\bar{F}^{\theta}_{\mu,\sigma}(x) = e^{-x/\theta e^{\mu}}, x > 0.$$

Then their sum follows an Erlang distribution, satisfying:

$$\mathbb{P}^{\theta}_{\mu,\sigma}[\,\textstyle\sum_{2}^{n} \mathbf{a}_{i,0} > n\beta\,] = \displaystyle\sum_{k=0}^{n-2} \frac{e^{-n\beta/(\theta e^{\mu})}}{k!} \left(\frac{n\beta}{\theta e^{\mu}}\right)^{k},$$

such that

$$\begin{aligned}\mathbb{P}^{\theta}_{\mu,\sigma}[\max_{2,\ldots,n} \mathbf{a}_{i,0} > n\beta\,] &\asymp (n-1)\mathbb{P}^{\theta}_{\mu,\sigma}[\,\mathbf{a}_{1,0} > n\beta\,] = (n-1)e^{-n\beta/(\theta e^{\mu})} \\ &\ll \sum_{k=0}^{n-2} \frac{e^{-n\beta/(\theta e^{\mu})}}{k!} \left(\frac{n\beta}{\theta e^{\mu}}\right)^{k} = \mathbb{P}^{\theta}_{\mu,\sigma}[\,\textstyle\sum_{i=2}^{n} \mathbf{a}_{i,0} > n\beta\,].\end{aligned}$$

and

$$\frac{\mathbb{P}^{\theta}_{\mu,\sigma}[\max_{2,\ldots,n} \mathbf{a}_{i,0} > n\beta\,]}{\mathbb{P}^{\theta}_{\mu,\sigma}[\,\sum_{i=2}^{n} \mathbf{a}_{i,0} > n\beta\,]} \asymp 0.$$

S2.1.5 Distributions with strong tail decay and classic accounts of the wisdom of crowds

It is instructive to investigate the behavior of the lower bound for light-tailed distributions as well. Sub-Gaussian distributions are a class of probability distribution with strong tail decay (at least as fast as a Gaussian). Suppose $\mathbf{x}$ is a random variable with mean $\mu + \theta$ and cumulative distribution $F^{\theta}_{\mu,\sigma}$. Furthermore, suppose that $\mathbf{x} - \mu - \theta$ is sub-Gaussain with variance-proxy

parameter σ, thereby, satisfying:

$$\bar{F}^{\theta}_{\mu,\sigma}(n\beta) = \mathbb{P}^{\theta}_{\mu,\sigma}[\mathbf{x} > n\beta] \leq e^{-(n\beta-\mu-\theta)^2/2\sigma^2}$$

On the other hand, we have $(1 + F^{\theta}_{\mu,\sigma}(n\beta) + F^{\theta}_{\mu,\sigma}(n\beta)^2 + \ldots + F^{\theta}_{\mu,\sigma}(n\beta)^{n-2}) \leq n - 1$, which we can combine with the above to get that

$$\begin{aligned} &F^{\theta}_{\mu,\sigma}(\beta)(1 - F^{\theta}_{\mu,\sigma}(n\beta)^{n-1}) \\ &= F^{\theta}_{\mu,\sigma}(\beta)(1 - F^{\theta}_{\mu,\sigma}(n\beta))(1 + F^{\theta}_{\mu,\sigma}(n\beta) + F^{\theta}_{\mu,\sigma}(n\beta)^2 + \ldots + F^{\theta}_{\mu,\sigma}(n\beta)^{n-2}) \\ &\leq nF^{\theta}_{\mu,\sigma}(\beta)\exp\left(-\frac{(n\beta-\mu-\theta)^2}{2\sigma^2}\right) \to 0, \text{ as } n \to \infty, \text{for any } \beta > 0. \end{aligned}$$

Therefore, there are no set of parameters μ and σ that lead to a non-trivial, asymptotic lower bound on Ω_n for random variables with sub-Gaussian tails: $\underline{\Omega}_n(\omega, \mathcal{F}^{\theta}_{\mu,\sigma}) \asymp 0$, for all θ, μ, σ. As an example, consider the folded Gaussian distribution, which is defined as the absolute value of a normally distributed random variable with mean θe^{μ} and variance σ:

$$F^{\theta}_{\mu,\sigma}(x) = \Phi\left(\frac{x - \theta e^{\mu}}{\sigma}\right) + \Phi\left(\frac{x + \theta e^{\mu}}{\sigma}\right) - 1, x > 0, \tag{S.21}$$

where Φ is the standard normal distribution. In Figure S1.D, bottom, we have plotted $\Omega_n(\omega, \mathcal{F}^{\theta}_{\mu,\sigma})$ with $\omega = 1/3$, $\theta = 2$, $n = 50$, and initial estimates following a folded-Gaussian distribution. There are no range of distribution parameters, μ and σ, for which Ω_n increases above 0.6. Indeed, for such light-tailed distributions, admitting finite first and second moments, we can show that the limiting expected absolute error of the collective estimate with centralization ω, $\mathbf{a}^n(\omega)$, is higher than the decentralized baseline, $\mathbf{a}^n(0)$.

Consider the case where $\mathcal{F}^{\theta}_{\mu,\sigma}$ admits the following finite first and second moments: $\mathbb{E}^{\theta}_{\mu,\sigma}[\mathbf{a}_{1,0}] = \theta e^{\mu}$, and $\mathbb{E}^{\theta}_{\mu,\sigma}[\mathbf{a}^2_{1,0}] = \theta^2 e^{2\mu} + \sigma^2$. Then $\mathbf{a}^n(0) \to \theta e^{\mu}$, and $\mathbf{a}^n(\omega) \to \omega\mathbf{a}_{1,0} + (1-\omega)\theta e^{\mu}$, both almost surely, as $n \to \infty$. Hence,

$$\mathbb{E}^{\theta}_{\mu,\sigma}[|\mathbf{a}^n(\omega) - \theta|] > |\mathbb{E}^{\theta}_{\mu,\sigma}[\mathbf{a}^n(\omega) - \theta]| = |\theta(1 - e^{\mu})| \asymp \mathbb{E}^{\theta}_{\mu,\sigma}[|\mathbf{a}^n(0) - \theta|].$$

We can repeat the same calculations for the expected mean squared errors as well:

$$\mathbb{E}^{\theta}_{\mu,\sigma}[(\mathbf{a}^n(0)-\theta)^2] \to \theta^2(1-e^{\mu})^2, \text{ and}$$
$$\mathbb{E}^{\theta}_{\mu,\sigma}[(\mathbf{a}^n(\omega)-\theta)^2] \to \theta^2(1-e^{\mu})^2 + \omega^2\sigma^2 > \theta^2(1-e^{\mu})^2 \asymp \mathbb{E}^{\theta}_{\mu,\sigma}[(\mathbf{a}^n(0)-\theta)^2].$$

For distributions with light tails decentralized networks outperform centralized ones, in expectation for absolute and squared errors, for any choice of parameters μ and σ. This verifies the classical accounts of the wisdom of crowds, whereby the law of large numbers guarantees almost sure convergence of the collective estimate for structures with vanishing individual influences [16, Proposition 2].

Indeed, among all convex combinations the simple average, $\mathbf{a}^n(0)$, has the minimum variance. Since all estimators in this class, $\mathbf{a}^n(\omega), \omega \geq 0$, have the same expected value, the simple average, $\mathbf{a}^n(0)$, is, in fact, the mean squared error (MSE) minimizer in this class. In this subsection, we point out that even though the variance/MSE of $\mathbf{a}^n(\omega)$ is minimum when $\omega = 0$, $\mathbf{a}^n(\omega)$, with $\omega > 0$ fixed, can "often" outperform $\mathbf{a}^n(0)$, i.e., fall closer to the truth, θ. We show this by lower bounding the probability, Ω_n, that $|\mathbf{a}^n(\omega) - \theta| < |\mathbf{a}^n(0) - \theta|$. Subsequently, we identify heavy-tailedness conditions that make this event likely and Ω_n large.

It is worth highlighting that although for $\mu = 0$, the sample mean (simple average) is a minimum-variance, unbiased estimator (MVUE); in statistics, it is well-known that simple average is not "optimal" for closeness in many situations. For instance, in the presence of outliers or extreme values the sample median is preferred to sample mean due to its robustness properties [17]. Our calculations in Subsections S2.1.1-S2.1.4 are of a similar flavor, pointing out the superiority of a weighted average when the underlying distributions are heavy-tailed.

In subsection S2.2, we discuss direct numerical simulation of the value of Ω_n for various distribution classes. In sub-subsection S2.2.1, we identify other comparable right-skewness conditions for making Ω_n large, e.g., greater than $1/2$, by analyzing the location of the medians of the two estimators, $\mathbf{a}^n(\omega), \omega > 0$ and $\mathbf{a}^n(0)$. In section S3, we show that the feature, Ω, that we identify from this theory has significant explanatory power for determining whether experimentally measured collective estimation outcomes improve, after group members interact with each other.

S2.2 Numerical simulations

For numerical simulations, we have fixed $\theta = 2$, $\omega = 1/3$, and $n = 50$. The choice of $\omega = 1/3$ is arbitrary and our conclusions remain valid for $\omega > 0$, as verified by the robustness checks in section S4. This choice is motivated by our observation in subsection S1.1 that ω for a star network converges to $1/3$ as $n \to \infty$. This also allows us to juxtapose our simulations with common experimental setups that use the star topology as archetypal of centralized structures [7].

Note that with $\omega = 1/3$ fixed, the dependence of Ω_n on the network structure is removed. Therefore, $\Omega_n(\omega, \mathcal{F}^{\theta}_{\mu,\sigma})$ is entirely determined by the distribution of the initial estimates, $\mathcal{F}^{\theta}_{\mu,\sigma}$, i.e. the estimation context. Here, we study our proposed task feature, Ω_n, numerically for a palette of empirically relevant distributions.

For any distribution of the initial estimates, $\mathcal{F}^{\theta}_{\mu,\sigma}$, and number of agents, n, we calculate Ω_n using a Monte Carlo method. We sample n initial estimates and calculate the collective estimates, $\mathbf{a}^n(1/3)$ and $\mathbf{a}^n(0)$, using equation (S.5). If $\mathbf{a}^n(1/3)$ is closer to the truth, θ, than $\mathbf{a}^n(0)$, implying that a centralized network performed better than a decentralized network, then we add to our tally of Ω_n. We repeat this procedure N times, where N is large enough to allow for the value of Ω_n to converge (see the simulation procedure 1). The results in Figure S1 are obtained in this manner with $\theta = 2$, $n = 50$, and $N = 10,000$ for four different distributions: Pareto (S.10), log-Laplace (S.14), log-normal (S.16), and folded-Gaussian (S.21).

Procedure 1: $\Omega_n(\omega, \mathcal{F}^{\theta}_{\mu,\sigma})$ computation

---

Input: $\mathcal{F}_{\theta}(\mu, \sigma)$, n, ω, N
Output: $\Omega_n(\omega, \mathcal{F}^{\theta}_{\mu,\sigma})$
Initialize $\Omega_n(\omega, \mathcal{F}^{\theta}_{\mu,\sigma}) = 0$.
for $j = 1 : N$ **do**
  Sample $\mathbf{a}_{1,0}, \ldots, \mathbf{a}_{n,0} \sim \mathcal{F}_{\theta}(\mu, \sigma)$.
  Compute $\mathbf{a}^n(0)$ and $\mathbf{a}^n(\omega)$ according to (S.5).
  if $|\mathbf{a}^n(\omega) - \theta| < |\mathbf{a}^n(0) - \theta|$ **then**
    Update $\Omega_n(\omega, \mathcal{F}^{\theta}_{\mu,\sigma}) = \Omega_n(\omega, \mathcal{F}^{\theta}_{\mu,\sigma}) + 1/N$.
  end
end

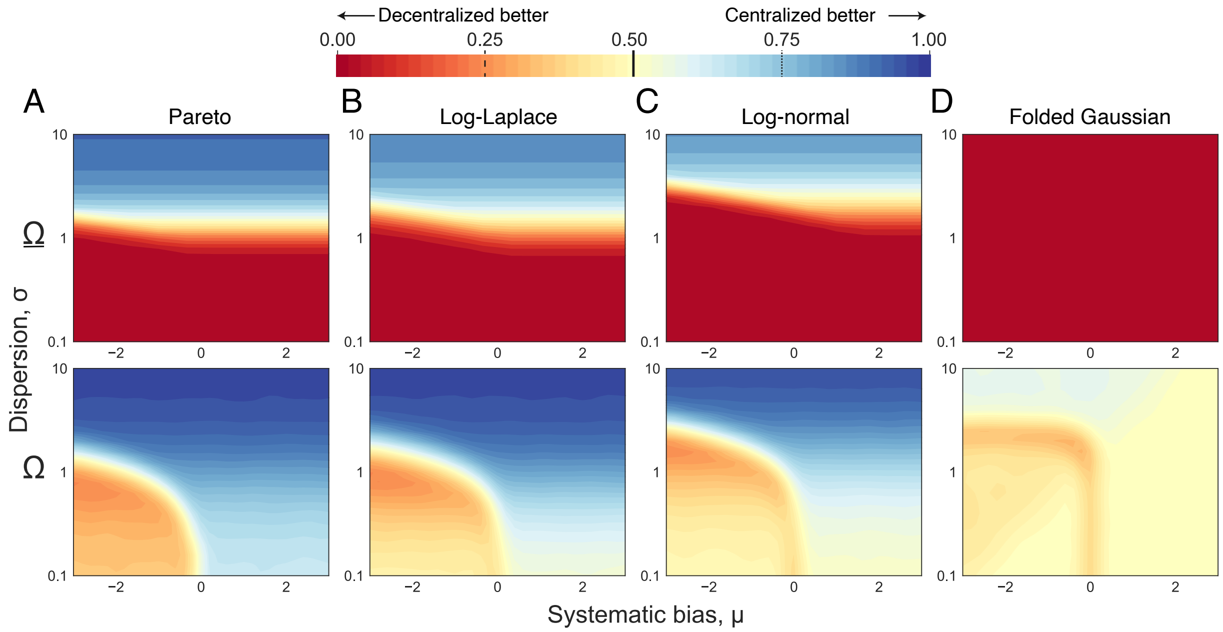

Figure S1: Simulating the lower bound $\underline{\Omega}_n(\omega, \mathcal{F}^{\theta}_{\mu,\sigma})$ and the actual value $\Omega_n(\omega, \mathcal{F}^{\theta}_{\mu,\sigma})$, under different distributions for the initial estimates: Pareto (S.10), log-Laplace (S.14), log-normal (S.16), and folded Gaussian (S.21). In all the plots, we have fixed $\omega = 1/3$, $\theta = 2$, and $n = 50$.

S2.2.1 The effect of the systematic bias, μ

To see the effect of the log-normal distribution parameters, μ and σ, in a different light, it is instructive to study the behavior of the median of the random variable $\mathbf{a}^n(\omega)$. In particular, we are interested in the location of Median$[\mathbf{a}^n(\omega)]$ with respect to the truth θ, as the distribution parameter μ is varied. We do so in the limit of large group sizes, $n \to \infty$. Note that since log-normal distributions have finite moments, the strong law of large numbers applies. Hence, as $n \to \infty$, $\mathbf{a}^n(0)$ converges almost surely to

$$\mathbb{E}[\mathbf{a}_{i,0}] = \exp(\log\theta + \mu + \sigma^2/2)$$

In particular we also have that

$$\lim_{n\to\infty} \text{Median}[\mathbf{a}^n(0)] = \exp(\log\theta + \mu + \sigma^2/2).$$

On the other hand, note that for $\omega > 0$, $\mathbf{a}^n(\omega) = \omega\mathbf{a}_{1,0} + (1-\omega)\mathbf{a}^n(0)$. Hence, as $n \to \infty$, $\mathbf{a}^n(\omega) \to \omega\mathbf{a}_{1,0} + \exp(\log\theta + \mu + \sigma^2/2)$, almost surely. Therefore,

$$\begin{aligned}\lim_{n\to\infty} \text{Median}[\mathbf{a}^n(\omega)] &= \omega\,\text{Median}[\mathbf{a}_{i,0}] + (1-\omega)\exp(\log\theta + \mu + \sigma^2/2) \\ &= \omega\exp(\log\theta + \mu) + (1-\omega)\exp(\log\theta + \mu + \sigma^2/2),\end{aligned}$$

where we have used the fact that $\text{Median}[\mathbf{a}_{i,0}] = \exp(\log\theta + \mu)$. Next, we note that depending on where the distributional parameters, θ, σ, and μ, are located, three cases may arise:

- If $\exp(\log\theta+\mu+\sigma^2/2) < \theta$, or equivalently, $\mu < -\sigma^2/2$, then $\lim_{n\to\infty}$ $\text{Median}[\mathbf{a}^n(\omega)] < \lim_{n\to\infty}$ $\text{Median}[\mathbf{a}^n(0)] < \theta$. In this case, as $n \to \infty$, at least half of the time, $\mathbf{a}^n(0)$ falls closer to θ than $\mathbf{a}^n(\omega)$, hence, $\lim_{n\to\infty} \Omega_n < 1/2$; see Figure S2.A.

- If $\exp(\log\theta + \mu) < \theta < \exp(\log\theta + \mu + \sigma^2/2)$, or equivalently, $-\sigma^2/2 < \mu < 0$, then $\lim_{n\to\infty}$ $\text{Median}[\mathbf{a}^n(\omega)] < \theta < \lim_{n\to\infty}$ $\text{Median}[\mathbf{a}^n(0)]$. In this case, the limiting value of Ω_n may be less then, or greater than, but is close to $1/2$; see Figure S2.B.

- If $\theta < \exp(\log\theta+\mu+\sigma^2/2)$, or equivalently, $0 < \mu$, then $\theta < \lim_{n\to\infty}$ $\text{Median}[\mathbf{a}^n(\omega)] < \lim_{n\to\infty}$ $\text{Median}[\mathbf{a}^n(0)]$. In this case, as $n \to \infty$, at least half of the time, $\mathbf{a}^n(\omega)$ falls closer to θ than $\mathbf{a}^n(0)$, hence, $\lim_{n\to\infty} \Omega_n > 1/2$; see Figure S2.C.

Finally, it is worth noting that a similar argument applies to any right-skewed and heavy-tailed distribution, for which the population mean exists and is greater than the population median.

S3 Empirical analysis of estimation contexts in prior work

To empirically illustrate the explanatory power of this theory, we use data from four published experiments [8, 18, 7, 6], in which $2,885$ participants organized into 99 independent groups completed a total of 54 estimation tasks generating $15,562$ individual estimations and 687 collective estimations. Each task induces a different distribution on the initial estimates that are measured empirically. Therefore, each task constitutes an estimation context in our framework and we have a total of 54 estimation contexts.

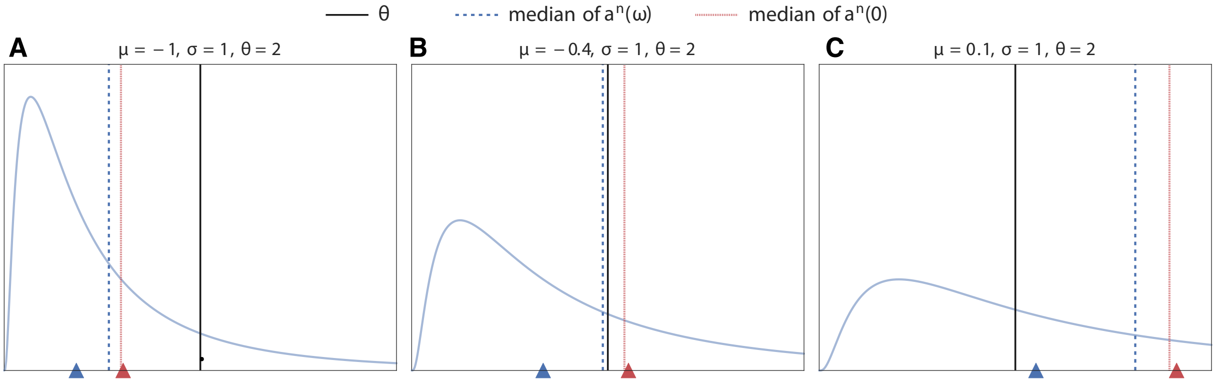

Figure S2: Simulating the medians of $\mathbf{a}^n(\omega)$ and $\mathbf{a}^n(0)$ for three different values of the systematic bias, μ. The distribution median, marked in blue on the x-axis, lags the distribution mean, marked in red. Subsequently, the median of $\mathbf{a}^n(\omega)$ is always less than the median of $\mathbf{a}^n(0)$ for the distributions studied here. In this framing, there are three different levels of bias: panel **A**, when the distribution of initial estimates significantly under-estimates the truth, $\mu < -\sigma^2/2$, then the median of $\mathbf{a}^n(0)$ is closer to the truth, θ, than the median of $\mathbf{a}^n(\omega)$, in this case, $\Omega_n < 1/2$; panel **B**, when the distribution of initial estimates slightly under-estimates the truth, $-\sigma^2/2 < \mu < 0$, then the truth lies between the medians of $\mathbf{a}^n(\omega)$ and $\mathbf{a}^n(0)$, and $\Omega \approx 0.5$; panel **C**, when the distribution of initial estimates over-estimates the truth, $\mu > 0$, then the median $\mathbf{a}^n(\omega)$ is closer to the truth, leading to $\Omega_n > 1/2$. In these simulations, we have fixed $\omega = 1/3$, $\theta = 2$, $n = 50$, and $N = 10,000$, where N is the number of samples used to simulate the median values numerically.

S3.1 Regression Analysis

Our main empirical analyses, shown in Figure 3 and Table S1, are based on a mixed effect model with a random effect to account for the nested structure of the data.

In particular, the logistic regression equation for Figure 3.C (Table S1, Model 1) is:

$$y_{ij} = \frac{1}{1 + \exp(b_0 + b_1 R_j + v_i + v_k + \epsilon_{ijk})},$$

where y_{ij} is a binary indicator of whether the i-th group on the j-th task improved its collective estimate after social interaction, b_0 is the fixed intercept, b_1 is the fixed coefficient for our proposed feature of the estimation context, v_i is the random coefficient for the i-th group, v_k is the random coefficient for the k-th experiment, and ϵ_{ijk} is a Gaussian error term.

The regression equation for Figure 3.D (Table S1, Model 2) is:

$$y_{ij} = b_0 + b_1 R_j + b_2 I_i + b_3 I_i R_j + v_i + v_k + \epsilon_{ijk},$$

where y_{ij} is the standardized (z-score) absolute error of the revised collective estimate for the i-th group in the j-th estimation context, b_0 is the fixed intercept for the regression model, b_1 is the fixed coefficient for our proposed feature of the estimation context, $I_i \in \{0, 1\}$ is an indicator variable of whether social interaction has occurred or not, b_2 is the fixed coefficient for the social influence centralization, b_3 is the fixed coefficient for the interaction term between our proposed feature of the estimation context and influence centralization, v_i is the random coefficient for the i-th group, and v_k is the random coefficient for the k-th experiment. Finally, ϵ_{ijk} is a Gaussian error term.

S3.2 Regression Analysis Experiment-by-Experiment

In addition to our main regression analyses outlined above, we further analyzed the empirical data with data subsets. Specifically, we re-fit the main regression models outlined in S3.1 to the data from each experiment (or study) individually. Whereas aggregating the data from four published experiments [9, 10, 14, 16] allowed us conduct our analyses with higher statistical power across a wider range of the R parameter space, analyzing the data experiment-by-experiment allows us to generate a range of coefficients to further understand potential variability across experiment implementations (albeit with lower statistical power and narrower ranges of R; see Panels A and B in Figure 3 in the main text). In doing so, we indeed observe variation in both, Model 1, the degree to which R explains the probability that a group improves after centralized social interaction [Lorenz et al. (2011), z-statistic $= 0.03$, $p = 0.973$; Gürçay et al. (2015), z-statistic $= 4.55$, $p < 0.001$; Becker et al. (2017), z-statistic $= 0.66$, $p = 0.510$; Becker et al. (2019), z-statistic $= -2.25$, $p = 0.025$], and, Model 2, the effect of the interaction between the centralization of the influence and R on the absolute error of the revised collective estimate [Lorenz et al. (2011), $b = -2.50$, t-statistic $= -0.62$, $p = 0.540$; Gürçay et al. (2015), $b = -20.52$, t-statistic $= -8.26$, $p < 0.001$; Becker et al. (2017), $b = 2.00$, t-statistic $= 0.28$, $p = 0.779$; Becker et al. (2019), $b = 6.76$; t-statistic $= 2.04$, $p < 0.043$].

Table S1: The main effects of task environment (relative log likelihood, R) and the interaction with social influence (i.e., centralization) Each datapoint is an experimental trial. The results are from a mixed effect model with a random effect for the group. Tables S3-S2 for robustness to these choices.

	Dependent variable:	
	Group improved after social interaction	Standardized Absolute Error
	generalized linear mixed-effects	*linear mixed-effects*
	(Model 1)	(Model 2)
R	4.924***	4.178***
	(1.183)	(1.164)
Social Influence $\in \{0,1\}$		2.470***
		(0.732)
R x Social Influence		−4.889***
		(1.250)
Intercept	−2.448***	−2.122***
	(0.711)	(0.683)
Observations	582	687
Log Likelihood	−382.664	−934.516
Akaike Inf. Crit.	773.329	1,883.033
Bayesian Inf. Crit.	790.795	1,914.759
Note:		*p<0.1; **p<0.05; ***p<0.01

S4 Robustness checks

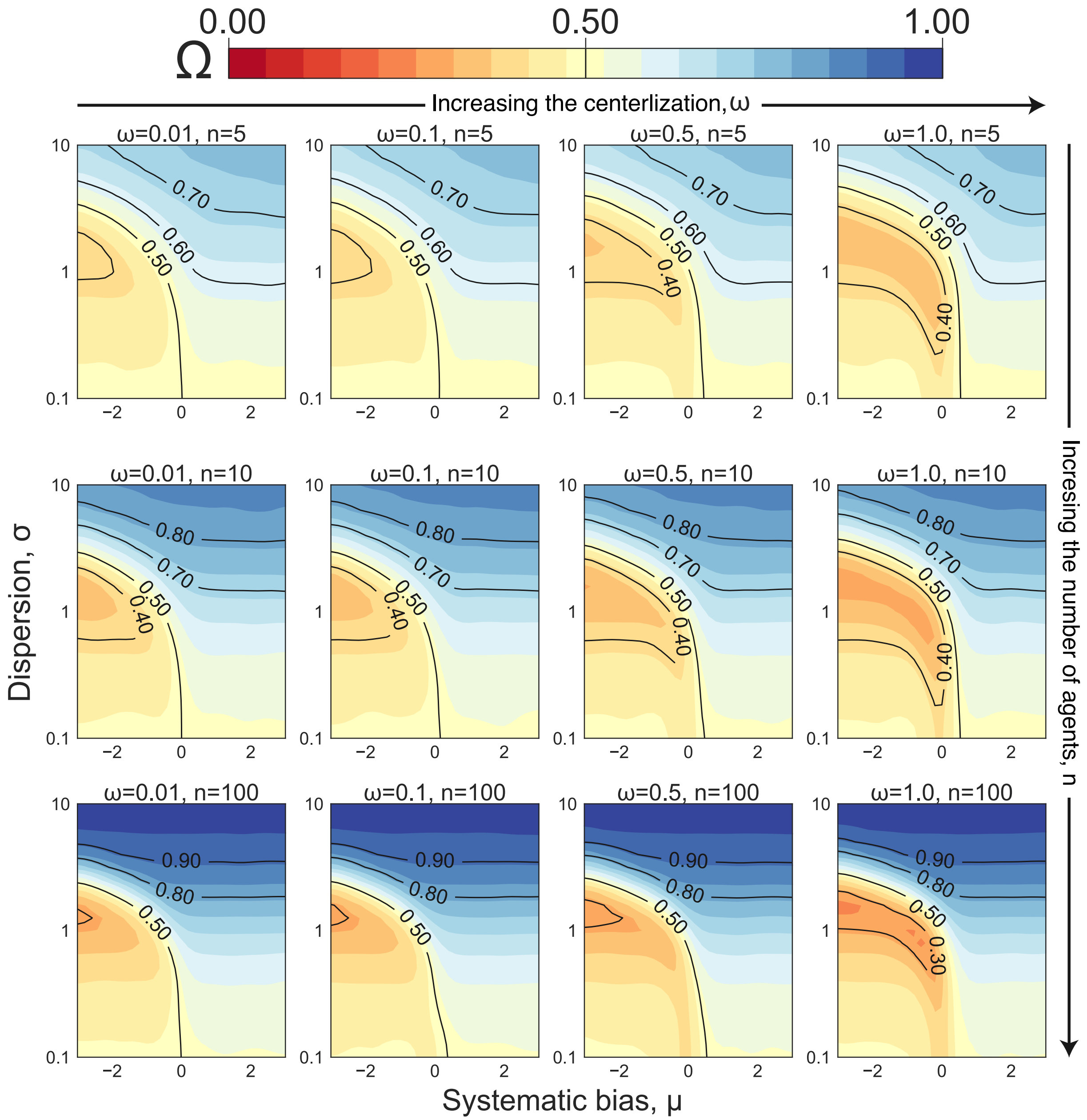

Figure S3: Robustness checks of the simulation results by varying ω and n when calculating our proposed feature of the estimation context, Ω, for the log-normal distribution. We find that the qualitative behavior of the phase diagram is robust to these changes. Increasing n or ω leads to sharper transitions from low Ω to high Ω.

Table S2: Robustness checks for Model 1 (by varying the task environment metric, the independent variable *R*, derived from the empirical data) for the effect of the task environment on group performance after social interaction. Each datapoint is an experimental trial. Note that for the Hill estimate of the tail index, the average estimate across all possible thresholds was used as the IV, and lower estimates indicate heavier tails. The results are from a mixed effect model with a random effect for the group. We find that the nature of the results is robust across alternatives metrics.

	Dependent variable:		
	Whether the group improved after social interaction		
	Kurtosis	*Skewness*	*Hill Estimate*
R	0.0002	0.026^{**}	-0.168^{***}
	(0.0005)	(0.012)	(0.047)
Intercept	0.364	0.120	0.834^{***}
	(0.282)	(0.236)	(0.287)
Observations	582	582	582
Log Likelihood	−391.395	−389.084	−384.547
Akaike Inf. Crit.	790.790	786.169	777.094
Bayesian Inf. Crit.	808.256	803.635	794.560
Note:			*p<0.1; **p<0.05; ***p<0.01

Table S3: Robustness checks for Model 2 (by varying the task environment metric, the independent variable *R*, derived from the empirical data) for the marginal effect of the interaction term between the centralization of influence and task environment on group performance—in terms of standardized absolute error. Each datapoint is an experimental trial. Note that for the Hill estimate of the tail index, the average estimate across all possible thresholds was used as the IV, and lower estimates indicate heavier tails. The results are from a mixed effect model with a random effect for the group and a random effect for the experiment/study.

	Dependent variable:		
	Standardized Absolute Error		
	Kurtosis	*Skewness*	*Hill Estimate*
	(1)	(2)	(3)
R	−0.0001	0.005	−0.215***
	(0.0002)	(0.008)	(0.052)
Social Influence $\in \{0,1\}$	−0.395***	−0.283**	−0.937***
	(0.124)	(0.142)	(0.163)
R x Social Influence	0.0001	−0.008	0.245***
	(0.0003)	(0.009)	(0.055)
Constant	0.338***	0.246*	0.801***
	(0.114)	(0.131)	(0.151)
Observations	687	687	687
Log Likelihood	−958.757	−951.470	−938.483
Akaike Inf. Crit.	1,931.514	1,916.940	1,890.966
Bayesian Inf. Crit.	1,963.240	1,948.666	1,922.693
Note:			*p<0.1; **p<0.05; ***p<0.01

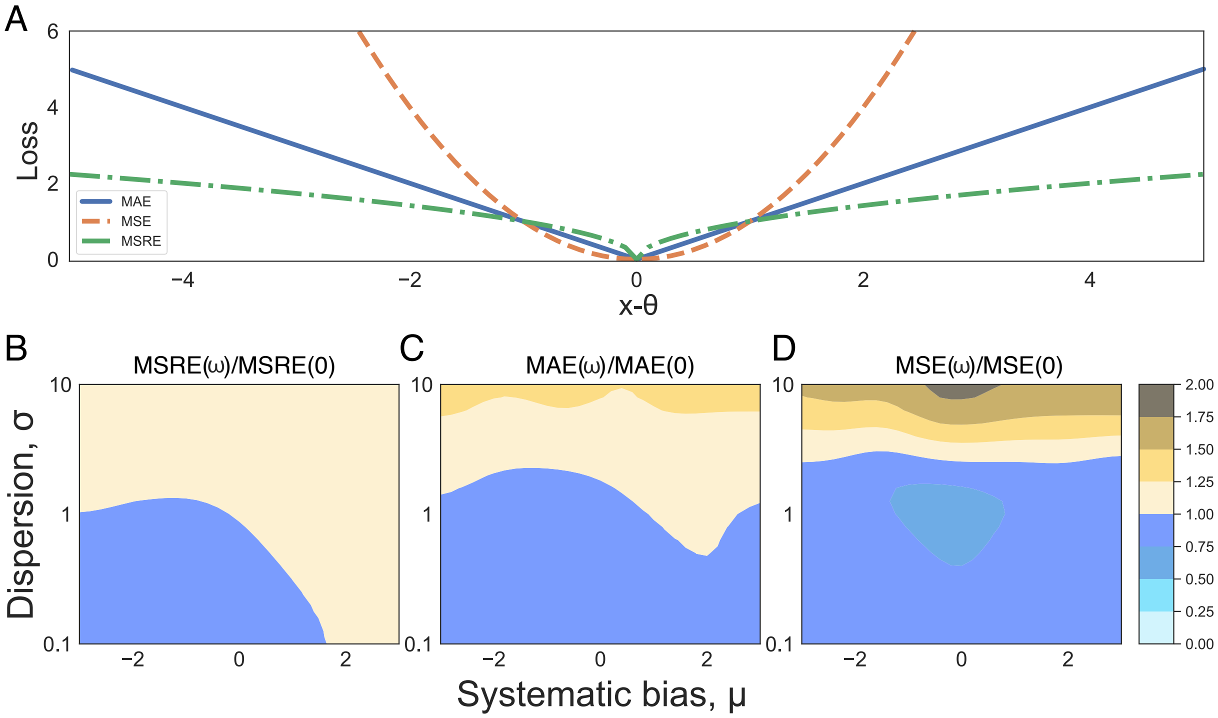

Figure S4: Panel **A** shows the loss as a function of error for the Mean Squared Error (MSE), Mean Absolute Error (MAE), and the Mean Square Root Error (MSRE) loss functions — see (S.6). Panels **B**, **C**, and **D** show the three loss functions for different values of μ and σ for a log-normal distribution. In each case, we plot the ratio of the loss function evaluated in a centralized structure, $\omega > 0$, over a decentralized structure $\omega = 0$. A ratio less than 1 indicates that the centralized network performs better than the decentralized network. The performance of the two influence structures can vary significantly as a function of the selected loss function. The choice of the loss function is typically application-dependent. For instance, if the reward for 'getting it right' is greater than the cost of being frequently wrong — as in domains where the loss and payoff are asymmetric, unbounded, or have a remote boundary [13, 19] — then the decentralized influence structure is more desirable when the dispersion is high. The initial estimates in these simulations are sampled from a log-normal distribution for a fixed number of agents ($n = 50$) and centralization level ($\omega = 1/3$).

S5 References

[1] N. E. Friedkin, A formal theory of social power, *Journal of Mathematical Sociology* **12**, 103 (1986).

[2] J. R. French Jr, A formal theory of social power, *Psychological review* **63**, 181 (1956).

[3] F. Harary, *Studies in Social Power* (University of Michigan, 1959), pp. 168–182. D. Cartwright, Ed.

[4] M. H. DeGroot, Reaching a consensus, *Journal of American Statistical Association* **69**, 118 (1974).

[5] E. Seneta, *Non-negative matrices and Markov chains* (Springer, 2006).

[6] J. Becker, E. Porter, D. Centola, The wisdom of partisan crowds, *Proceedings of the National Academy of Sciences* **116**, 10717 (2019).

[7] J. Becker, D. Brackbill, D. Centola, Network dynamics of social influence in the wisdom of crowds, *Proceedings of the National Academy of Sciences* **114**, E5070 (2017).

[8] J. Lorenz, H. Rauhut, F. Schweitzer, D. Helbing, How social influence can undermine the wisdom of crowd effect, *Proc. of the National Academy of Sciences* **108**, 9020 (2011).

[9] A. Almaatouq, *et al.*, Adaptive social networks promote the wisdom of crowds, *Proceedings of the National Academy of Sciences* **117**, 11379 (2020).

[10] B. Jayles, *et al.*, The impact of incorrect social information on collective wisdom in human groups, *arXiv preprint arXiv:2003.06160* (2020).

[11] B. Jayles, *et al.*, How social information can improve estimation accuracy in human groups, *Proceedings of the National Academy of Sciences* **114**, 12620 (2017).

[12] A. B. Kao, *et al.*, Counteracting estimation bias and social influence to improve the wisdom of crowds, *Journal of The Royal Society Interface* **15**, 20180130 (2018).

[13] M. S. Lobo, D. Yao, Human judgement is heavy tailed: Empirical evidence and implications for the aggregation of estimates and forecasts, *INSEAD Working Paper No. 2010/48/DS. Available at SSRN: 1638811* .

[14] P. Mavrodiev, C. J. Tessone, F. Schweitzer, Quantifying the effects of social influence, *Scientific reports* **3**, 1360 (2013).

[15] J. Nair, A. Wierman, B. Zwart, *The fundamentals of heavy tails: Properties, emergence, and estimation*, vol. 53 (Cambridge University Press, 2022).

[16] B. Golub, M. O. Jackson, Naive learning in social networks and the wisdom of crowds, *American Economic Journal: Microeconomics* **2**, 112 (2010).

[17] P. J. Bickel, On some robust estimates of location, *The Annals of Mathematical Statistics* **36**, 847 (1965).

[18] B. Gürçay, B. A. Mellers, J. Baron, The power of social influence on estimation accuracy, *Journal of Behavioral Decision Making* **28**, 250 (2015).

[19] L. Fang, A. Yasuda, The effectiveness of reputation as a disciplinary mechanism in sell-side research, *The Review of Financial Studies* **22**, 3735 (2009).
